# Association between heart failure and the incidence of cancer: a systematic review and meta-analysis

**DOI:** 10.1093/ehjopen/oead073

**Published:** 2023-08-03

**Authors:** Vikash Jaiswal, Song Peng Ang, Vibhor Agrawal, Maha Hameed, Marina Raouf Abdelmessih Saleeb, Akash Jaiswal, Maitri Shah, Nicole Mae Lao, Jia Ee Chia, Kusum Paudel, Alessia Gimelli, Jerome Zacks

**Affiliations:** Department of Cardiovascular Research, Larkin Community Hospital, South Miami, FL 33143, USA; JCCR Cardiology Research, Varanasi, India; Department of Internal Medicine, Rutgers Health/Community Medical Center, Toms River, NJ, USA; Department of Medicine, King George’s Medical University, Lucknow, India; Department of Internal Medicine, Florida State University/Sarasota Memorial Hospital, Sarasota, FL, USA; Public Health Institute, Faculty of Health, Liverpool John Moores University, Liverpool L2 2QP, UK; Department of Geriatric Medicine, All India Institute of Medical Science, New Delhi, India; Department of Cardiovascular Research, Larkin Community Hospital, South Miami, FL 33143, USA; Department of Medicine, Medical College of Wisconsin, Milwaukee, WI 53226, USA; Department of Internal Medicine, Texas Tech University Health Science Center, El Paso, TX, USA; Department of Medicine, Kathmandu University School of Medical Sciences, Panauti 45209, Nepal; Department of Imaging, Fondazione Toscana ‘Gabriele Monasterio’, via Moruzzi n.1, Pisa 56124, Italy; Department of Cardiology, The Icahn Medical School at Mount Sinai, New York, NY 10128, USA

**Keywords:** Heart failure, Malignancy, Cancer, Outcomes

## Abstract

**Aims:**

The association between heart failure (HF) patients and the incidence of cancer is not well understood, with conflicting results to date. The aim of this meta-analysis was to evaluate whether patients with HF have a higher risk of developing cancer.

**Methods and results:**

We performed a systematic literature search using PubMed, Embase, and Scopus for relevant articles from inception until 10 December 2022. The primary clinical outcome was the incidence of cancer. Secondary endpoints were the incidence of breast cancer, lung cancer, haematological cancer, colorectal cancer, and prostate cancer. A total of 9 articles with 7 329 706 (515 041 HF vs. 6 814 665 non-HF) patients were involved in the analysis. The mean age of the patients in the HF and the non-HF groups was 69.06 and 66.76 years. The median follow-up duration was 6.7 years. The most common comorbidity among both groups includes diabetes mellitus (27.58 vs. 14.49%) and hypertension (81.46 vs. 57.38%). Patients with HF were associated with a significant increase in the incidence of cancer {hazard ratio [HR], 1.43 [95% confidence interval (CI): 1.21–1.68], *P* < 0.001}, breast cancer [HR, 1.28 (95% CI: 1.09–1.50), *P* < 0.001], lung cancer [HR, 1.89 (95% CI: 1.25–2.85), *P* < 0.001], haematological cancer [HR, 1.63 (95% CI: 1.15–2.33), *P* = 0.01], and colorectal cancer [HR, 1.32 (95% CI: 1.11–1.57), *P* < 0.001] compared with patients without HF. However, the incidence of prostate cancer was comparable between both groups [HR, 0.97 (95% CI: 0.66–1.43), *P* = 0.88].

**Conclusion:**

This meta-analysis confirms that the state of HF is associated with a higher risk for incident cancer. These data may aid in raising awareness with physicians that cancer may develop in patients with prevalent heart failure and that early screening and evaluation may be useful in an early diagnosis of cancer.

## Graphical Abstract

**Graphical Abstract:**
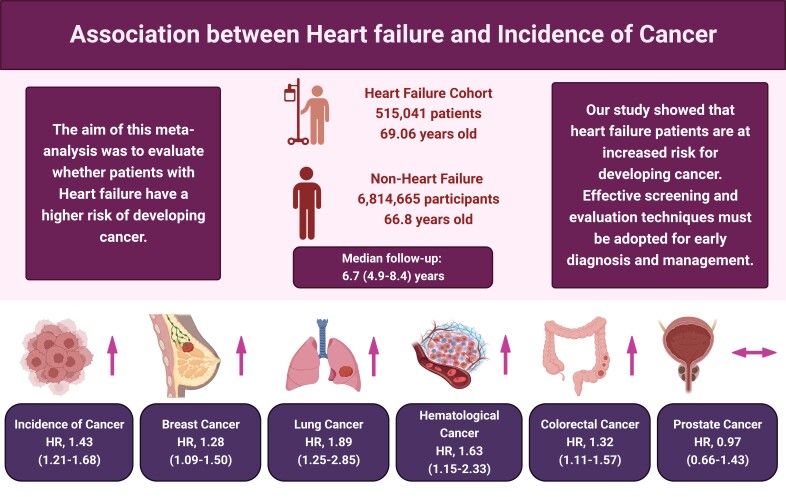
Central illustration highlighting the association between heart failure and cancer incidence.

## Introduction

Cancer is the second leading cause of death globally, accounting for 21% of all deaths, following heart disease.^1^ Over the past decade, new cancer cases have risen by 26.3% from 18.7 million in 2010 to 23.6 million in 2019, with a corresponding 20.9% increase in global cancer mortality from 8.29 to 10 million during the same period.^2^ Compared with these cancer statistics, the global burden of heart failure (HF) consists of a prevalence of 1–3% in the general adult population, an incidence of 1–20 cases per 1000 population, and 5-year mortality of 50–75%.^3^ The lifetime risk of HF ranges between 20 and 45% after the age of 45, with trends of increasing incidence of HF with preserved ejection fraction (HFpEF) and decreasing incidence of HF with reduced ejection fraction (HFrEF).^4^

Cardiovascular disease (CVD) and cancer share several risk factors, including smoking, obesity, and dyslipidaemia, and non-modifiable factors such as age, sex, and genetic mutations, particularly within the Wnt/b-catenin pathway and dual-specificity tyrosine phosphorylation-regulated kinase 1B.^5–7^ Studies suggest that patients with CVD have a higher likelihood of developing cancer, possibly due to two main hypotheses: inflammation, oxidative stress, and genetic predisposition promoting both conditions or HF acting as an oncogenic state through neurohormonal activation of the renin-angiotensin-aldosterone system and secretion of SERPINA3, which supports tumour growth.^6^ Furthermore, inflammatory cytokines and metabolic aberrations in glucose oxidation, aerobic glycolysis, and *de novo* lipogenesis have been implicated in the connection between HF and cancer.^8,9^ Clonal haematopoiesis of undetermined significance due to somatic mutations and abnormal fibroblast proliferation are all shared characteristics between cancer and CVD.^8–10^

Because of these overlaps in metabolic pathways and clinical contributors to the development of cancer and HF, the possibility of a clinical correlation between the two is exigent, although a definitive association between the two is yet to be established. To mitigate this, reverse cardio-oncology, i.e. addressing the risk of cancer in patients with CVD, is now on the rise.^10,11^ Aboumsallem *et al*.^10^ describe that this approach involves investigating the cardiovascular secretomes, inflammatory markers, and mutations in clonal haematopoiesis of undetermined significance through clinical prediction models aimed towards innovative management of cardiovascular patients to reduce cancer incidence. Additionally, reverse cardio-oncology aims to uncover novel therapeutic targets by gaining mechanical insights from *in vivo* and *in vitro* studies.^10^

Thus, the demand for early screening protocols to detect cancer in HF patients has grown, and reverse-oncology advocates for the documentation of oncology endpoints in cardiovascular trials and cardiovascular endpoints in oncology trials and the establishment of interdisciplinary research and clinical teams to facilitate the detection of cancer risk in cardiovascular patients and vice versa.^11^ Despite this, previous literature that aimed to determine the association between the two entities shows conflicting results. Hence, with the inclusion of recently published studies, we sought to perform a meta-analysis to determine whether there exists an association between HF and cancer risk.

## Methods

This meta-analysis was conducted and reported following the Preferred Reporting Items for Systematic review and Meta-Analysis 2020 guidelines and performed according to established methods, as described previously.^12–14^ The prespecified study protocol has been registered in the PROSPERO (CRD42022350346).

### Search strategy

We conducted a systematic literature search in PubMed, Embase, and Scopus using predefined MESH terms by using ‘AND’ and ‘OR’. The following search terms were used: ‘Heart failure’ OR ‘cardiac dysfunction’ OR ‘Myocardial Ischemia’ AND ‘Cancer’ OR ‘Malignancy’ OR ‘Neoplasm’. We queried databases from their search inception up until 10 December 2022 without any restrictions on the language of the studies. Search strategies are listed in Supplementary material online, *Table S1*.

All studies were carefully screened and exported to the Mendeley reference manager used to handle searched citations. A manual check was carried through to cross-check for any remaining duplicates. Two reviewers (V.J. and V.A.) reviewed the papers based on the title and abstract. Discrepancies regarding the inclusion of studies were arbitrated by another author (A.J.).

### Eligibility criteria

We included studies with adult patients ≥18 years of age. All prospective and retrospective cohort studies were sought to be eligible for inclusion in the study. It was decided to include studies with two arms in order to make a comparison between patients with and without HF. Selected studies compared patients with varying baseline characteristics and pathologies along with data for outcomes of interest.

Studies that were performed on animals, or reviews, case reports, case series, studies on patients <18 years, studies with a single arm or without HF patients, and studies without outcomes of interest were excluded from the review.

### Clinical outcomes

The primary outcome of this meta-analysis was the incidence of cancer. The secondary outcomes were incidences of breast cancer, lung cancer, haematological cancer, colorectal cancer, and prostate cancer.

### Data extraction and quality assessment

Two authors (V.A. and V.J.) extracted the following data: study type, author, study location, study follow-up duration, patient characteristics (number, age, gender, and comorbidities), and primary and secondary outcomes. We used the reported estimates when reported in the form of hazard ratios (HRs). If different estimates were available, we opted for HR with the most adjusted effect measure or propensity score-matched data where available. Two investigators (S.P.A. and J.E.C) independently appraised the potential risk of bias using the Newcastle–Ottawa scale (NOS) for observational studies.^15^ We then classified studies as low, moderate, or high quality based on the scores after evaluation.

### Statistical analysis

Statistical analysis was performed by calculating the HR using the random effect model, with a test for overall effect reported as *Z*-value, 95% confidence interval (CI), and probability value (*P*). Statistical significance was met if 95% CI does not cross numeric ‘1’ and *P* < 0.05. The heterogeneity among studies was assessed by Higgins’s *I*^2^ statistical model with *I*^2^ values. As a guide, *I*^2^ < 25% indicated low, 25–50% moderate, and >50% high heterogeneity.^16^ To test the possible effect of trials with a large sample size on the direction of clinical outcomes, a leave-one-out method was performed. Further sensitivity analyses using only adjusted estimates were carried out to assess the robustness of primary analyses. To explore the causes of heterogeneity of primary outcome, subgroup analyses were implemented based on study-level data, including follow-up period, study design, and sample size. Meta-regression was performed to look for potential effect modifiers for primary outcome using random effect models for study variance, and we included demographics and comorbidities in the interrogation of effect modifiers. Publication bias was assessed for outcomes with at least five studies using the graphical presentation of funnel plot asymmetry and quantitatively assessed by using Egger’s regression test.^17^ If publication bias was detected, the trim-and-fill method was employed to adjust for publication bias.^18^ All statistical analyses were performed using STATA version 17.1 (StataCorp, College Station, TX, USA).

## Results

### Baseline characteristics of patients in included studies

The initial search strategy yielded 581 articles, of which 75 duplicates were removed, and 431 articles were excluded after the title and abstract screening. The full-text review was performed on the remaining 75 studies, after which 65 studies were excluded from the final review and analysis for the following reasons: lack of appropriate comparison arm, wrong population, overlapped population, non-HF group, lack of follow-up data, or lack of outcome of interest (see Supplementary material online, *Figure S1*).

In summary, 10 studies were sought for the final review;^19–28^ however, 9 studies were included in the final analysis.^19–23, 25–28^ All of them were observational cohort studies, of which four were prospective^20,21,23,27^ and five were retrospective^19,22,25,26,28^ in study design. The total number of patients was 7 329 706, with 515 041 patients in the HF group and 6 814 665 in the non-HF group. The median follow-up duration was 6.7 (4.9–8.4) years. The mean age of patients among the HF and non-HF groups was 69.06 and 66.76 years, and the number of females in the HF and non-HF groups was 48 and 50.39%, respectively. The most common comorbidities among both groups of patients include hypertension (81.46 vs. 57.38%), diabetes mellitus (27.58 vs. 14.49%), and dyslipidaemia (47.27 vs. 33.11%) among HF groups of patients and non-HF groups. The study characteristics, patients’ demographics, and comorbidities are presented in *Table 1*. The quality assessment using NOS for observational studies showed that there was a low risk of bias across studies (see Supplementary material online, *Table S1*).

**Table 1:** Baseline demographics, comorbidities, and study characteristics of studies included in the meta-analysis

Study	Year published	Country	Study design	Study group	Sample size	Follow-up (years)	Age (years)	Female (%)	Hypertension (%)	DM (%)	Dyslipidaemia (%)	MI (%)	HR adjusted for
Kwak *et al*.^19^	2020	Korea	Retrospective	HF	128 441	4.06 (2.75–5.76)	67.1 ± 12.4	48.1	78.9	32.4	55.2	—	Age, sex, income, DM, smoking, alcohol, and BMI
				No HF	642 205		67.1 ± 12.4	48.1	55.0	19.4	34.5	—	
Hasin *et al*.^20^	2013	USA	Prospective	HF	596	7.7 ± 6.4	73 ± 14	53.0	67.0	20.0	29.0	21.0	BMI, smoking, and Charlson comorbidity index
				No HF	596		73 ± 14	53.0	54.0	8.0	30.0	6.0	
Hasin *et al*.^21^	2017	USA	Prospective	HF	228	4.9 ± 3.0	72 ± 14	54.0	81.0	28.0	66.0	100.0	Age, sex, and Charlson comorbidity index
				No HF	853		62 ± 15	37.0	61.0	16.0	61.0	100.0	
Banke *et al*.^22^	2016	Denmark	Retrospective	HF	9307	4.5 ± 2.3	67.8 ± 2.2	27.4	—	15.0	—	—	Gender, age, and date with follow-up time split into bands of 1-year
				No HF	4 959 275		—	—	—	—	—	—	
Selvaraj *et al*.^23^	2018	USA	Prospective	HF	1420	19.9 (11.0–26.8)	61 ± 9	0	42.2	10.2	36.5	2.3	Enrolment group, race, smoking, alcohol, aspirin use, family history of cancer, cirrhosis, proton pump inhibitor or H2 blocker use, sun exposure, any colonoscopy or sigmoidoscopy, physical exam, rectal exam, and prostate-specific antigen level tested
				No HF	26 921		55 ± 10	0	25.1	3.3	30.9	0.6	
Schwartz *et al*.^25^	2020	Denmark	Retrospective	HF	167 633	3.0 (1.6–7.8)	70.9 ± 13.3	45.0	—	17.0	—	25.0	Age, sex, baseline prevalence of ischaemic heart disease (including prior MI), DM, COPD, liver disease, and chronic kidney disease
				No HF	837 126	6.8 (3.1–11.8)	70.9 ± 13.3	45.0	—	7.0	—	5.0	
Roderburg *et al*.^26^	2021	Germany	Retrospective	HF	100 124	10	72.6 ± 12.2	54.0	—	37.4	—	—	NA
				No HF	100 124		72.6 ± 12.2	54.0	—	37.4	—	—	
Leedy *et al*.^27^	2021	USA	Prospective	HF	3272	8.4 (7.1–9.6)	68.1 ± 6.73	100	59.6	21.9	22.5	—	BMI, DM, smoking, primary care physician visit within 1-year, physical activity, alcohol, ethnicity, education, income, hormone use, hypertension, cardiac medication use, family history of cancer, history of cardiovascular disease, and high cholesterol
				No HF	143 545		—	100	—	—	—	—	
Bertero *et al*.^28^	2022	Italy	Retrospective	HF	104 020	5.6 (1.8–8.8)	—	—	85.84	31.33	38.47	16.45	NA
				No HF	104 020	5.3 (3.6–7.4)	—	—	79.95	28.04	24.51	3.43	

COPD, chronic obstructive pulmonary disease; BMI, body mass index; MI, myocardial infarction; NA, not available.

### Meta-analysis of the outcomes among patients with heart failure

**Figure 1:**
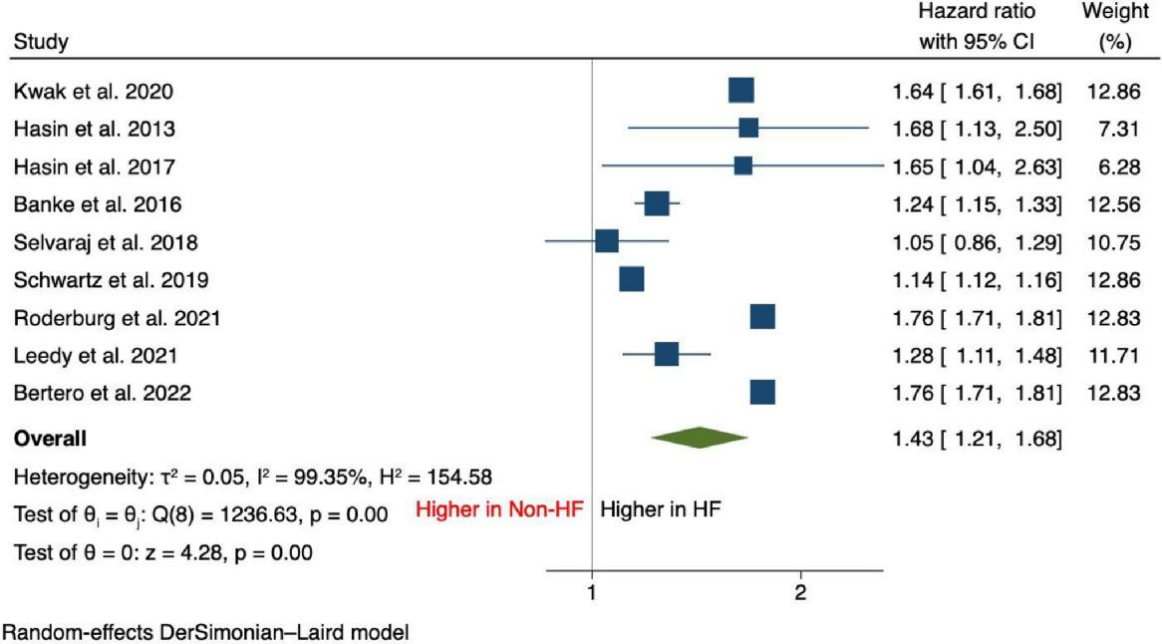
Forest plot of primary outcome—an overall incidence of cancer.

Patients with HF manifested a statistically significant increase in the incidence of cancer compared with patients without HF [HR, 1.43 (95% CI: 1.21–1.68), *P* < 0.001] (*Figure 1*).

**Figure 2:**
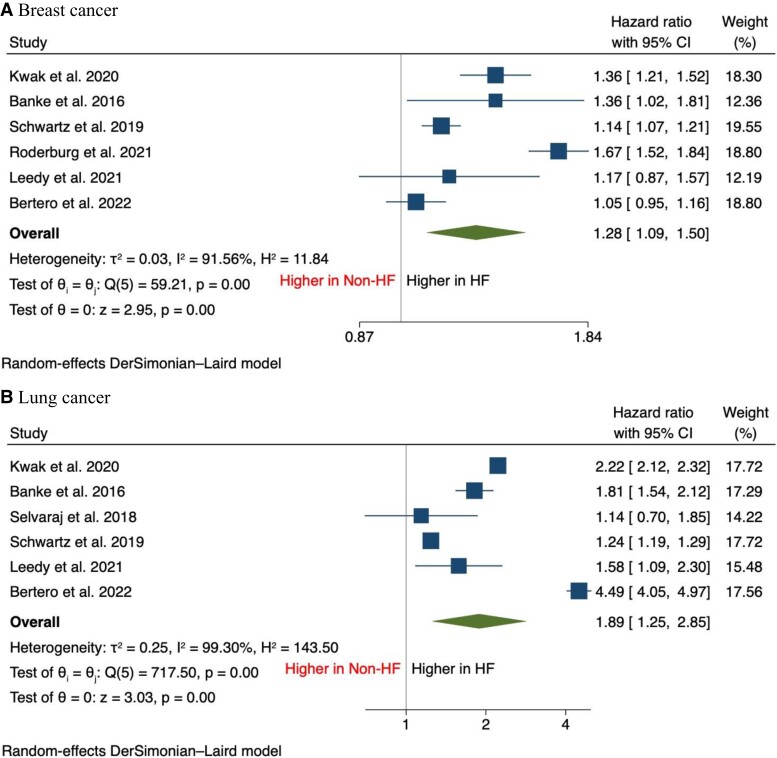
Forest plot of secondary outcomes including (*A*) breast cancer and (*B*) lung cancer.

**Figure 3:**
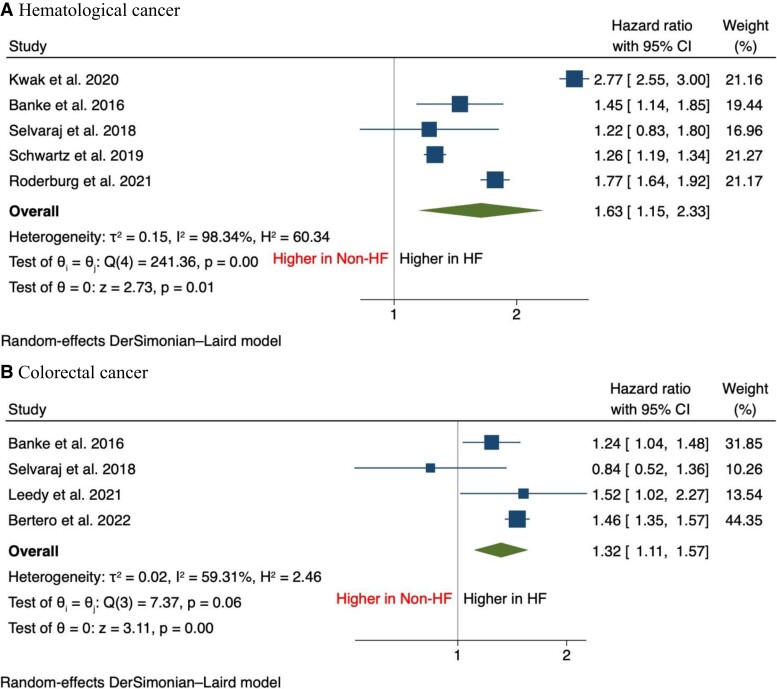
Forest plot of secondary outcomes including (*A*) haematological cancer and (*B*) colorectal cancer.

**Figure 4:**
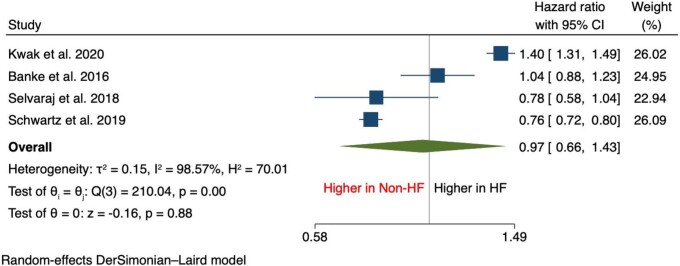
Forest plot of secondary outcomes—prostate cancer.

In terms of secondary outcomes, there was an increase in the incidence of breast cancer [HR, 1.28 (95% CI: 1.09–1.50), *P* < 0.001], lung cancer [HR, 1.89 (95% CI: 1.25–2.85), *P* < 0.001], haematological cancer [HR, 1.63 (95% CI: 1.15–2.33), *P* = 0.01], and colorectal cancer [HR, 1.32 (95% CI: 1.11–1.57), *P* < 0.001] compared with patients without HF (*Figures 2* and *3*). It was also found that the incidence of prostate cancer was comparable between both groups [HR, 0.97 (95% CI: 0.66–1.43), *P* = 0.88] (*Figure 4*).

### Sensitivity analysis

Sensitivity analyses were conducted for the incidence of overall cancer, the incidence of colon cancer, lung cancer, haematological cancer, breast cancer, and prostate cancer using the leave-one-out method and also using adjusted estimates where applicable. The results of the incidence of overall cancer remained unaltered after removing one study at a time, suggesting the robustness of the analysis (see Supplementary material online, *Figure S2*). Sensitivity analysis using adjusted estimates only showed that the incidence of cancer remained significantly higher among patients with HF compared with patients without HF [HR, 1.35 (95% CI: 1.08–1.69), *P* = 0.01] (see Supplementary material online, *Figure S3*).

Using only adjusted estimates, we found that the incidence of colorectal cancer remained significantly higher among the HF patients [HR, 1.28 (95% CI: 1.09–1.51), *P* < 0.01, *I*^2^ = 0%] (see Supplementary material online, *Figure S4*). However, it was noted that the results became non-significant after the removal of studies by Banke *et al*.^22^ and Bertero *et al*.^28^; thus, the influence of these studies on the results could not be excluded (see Supplementary material online, *Figure S5*). The incidence of breast cancer remained consistent with primary analysis after leave-one-out analysis and also using adjusted estimates, with patients with HF showing a higher incidence of breast cancer [HR, 1.24 (95% CI: 1.10–1.40), *P* < 0.001] (see Supplementary material online, *Figures S6* and *S7*). Lastly, the incidence of prostate cancer remained unaltered in terms of statistical significance even after excluding one study at a time (see Supplementary material online, *Figure S8*).

In terms of lung cancer, sensitivity analysis using adjusted estimates only showed a significant trend towards a higher incidence of lung cancer among HF patients [HR, 1.68 (95% CI: 1.13–2.49), *P* = 0.01] (see Supplementary material online, *Figure S9*). The leave-one-out analysis showed that results became non-significant after excluding the study by Kwak *et al*.,^19^ suggesting that results may be influenced by this study [HR, 1.80 (95% CI: 0.93–3.50), *P* = 0.08] (see Supplementary material online, *Figure S10*). Similarly, there was a non-significant trend of a higher incidence of haematological malignancy among HF patients using adjusted estimates only [HR, 1.72 (95% CI: 0.95–3.13), *P* = 0.08] (see Supplementary material online, *Figure S11*). However, results also became non-significant after removing the study by Roderburg *et al.*^26^ [HR, 1.59 (95% CI: 0.95–2.67), *P* = 0.08] (see Supplementary material online, *Figure S12*).

### Subgroup analysis

Subgroup analyses were conducted based on the study design, follow-up period, and sample size. Overall, it appeared that the incidence of cancer did not differ significantly between subgroups based on the stated covariates. Prospective studies [HR, 1.29 (95% CI: 1.07–1.57)] were shown to have a lower incidence of cancer among HF patients compared with that of retrospective studies [HR, 1.48 (95% CI: 1.21–1.82)], but subgroup difference was not evident (*P* = 0.34) (see Supplementary material online, *Figure S13*). In addition, no significant differences (*P* = 0.82) were found between studies with <5 years of follow-up [HR, 1.46 (95% CI: 1.15–1.87)] and studies with ≥5 years of follow-up [HR, 1.41 (95% CI: 1.12–1.78)] (see Supplementary material online, *Figure S14*). Lastly, the incidence of cancer among HF patients also did not differ significantly based on sample size (*P* = 0.77) (see Supplementary material online, *Figure S15*).

### Meta-regression

Meta-regression was conducted based on covariates, including age, gender, follow-up period, hypertension, hyperlipidaemia, and diabetes mellitus. Results of meta-regression showed that comorbidities, including diabetes mellitus (coefficient 1.63, *P* < 0.01), were potential positive effect modifiers of the incidence of cancer among HF patients (see Supplementary material online, *Figure S16*).

### Publication bias

Publication bias was assessed for the primary outcome and secondary outcomes meeting criteria as described in the Methods. There appeared to be no evidence of publication bias as evidenced by minimal funnel plot asymmetry as well as according to Egger’s regression test (*P* > 0.05) for incidence of overall cancer, breast cancer, lung cancer, and haematological cancer (see Supplementary material online, *Figures S17–S20*).

## Discussion

Our meta-analysis presents compelling evidence that HF patients display a significantly augmented incidence of cancer when compared with non-HF counterparts. The analysis indicates that HF patients possess a higher risk of contracting a number of cancers, including breast cancer, lung cancer, haematological cancer, and colorectal cancer. Conversely, the incidence of prostate cancer was found to be comparable between the two groups. Our sensitivity analyses attest to the robustness of the results, with the overall cancer incidence remaining significantly higher among HF patients. Our meta-regression analysis uncovered diabetes mellitus as a potential positive effect modifier of the incidence of cancer among HF patients. Moreover, subgroup analyses conducted on the basis of study design, follow-up period, and sample size did not reveal significant differences in the incidence of cancer among HF patients (*Table 2*).

**Table 2:** Meta-regression of potential effect modifiers for the primary outcome

Variables	Incidence of cancer	
	Coefficient	*P*
Demographics		
Age	0.021	0.21
Male	−0.264	0.38
Follow-up period	−0.021	0.33
Comorbidities		
DM	1.630	0.00

It is imperative to take into account the significant differences present in the individual studies comprising our meta-analysis. Of particular importance is the discrepancy in the duration of HF among the various studies. Notably, while a few studies^19,20,22,25^ included patients with newly diagnosed HF, others did not consider the duration of the disease. If the former was the case, it is plausible that the increased cancer risk noted in the HF group could be attributed to other factors associated with both HF and cancer rather than HF *per se*. On the other hand, neglecting to consider the duration of HF could result in an underestimation of cancer risk. Patients with longer durations of HF may have a greater susceptibility to cancer due to the progressive nature of the disease, which can engender changes in the body that heighten the risk of cancer over time, including chronic inflammation, immune system dysfunction, and oxidative stress.^8,11,29^

Another important aspect to consider is the meticulousness with which controls were matched for age, sex, comorbidities, and other pertinent variables. Although most of the studies included in the analysis employed matching procedures, the studies by Leedy *et al*.,^27^ Banke *et al*.,^22^ and Hasin *et al*.^20,21^ failed to do so. Properly matching controls is vital in minimizing confounding factors that may influence the association between HF and cancer risk. Furthermore, in addition to matching controls for confounding variables, the potential for surveillance bias also warrants consideration. In the context of HF, the use of comprehensive in-hospital examinations and specialized post-discharge care protocols may introduce surveillance bias, leading to potential impacts on the observed association between HF and cancer risk. To account for this possibility, Banke *et al*.^22^ and Kwak *et al*.^19^ employed a lag analysis, which excluded cases of cancer diagnosed within a specified time period following the diagnosis of HF (1 and 2 years, respectively). The findings of both studies indeed revealed a weaker, although still significant, association between cancer incidence and HF.^19,22^ The lack of a lag analysis in other studies included in our meta-analysis could have resulted in an overestimation of cancer incidence.

Another crucial aspect that differentiated the studies analysed pertains to their approach towards covariate adjustment. While all the studies presented unadjusted estimates for the HR, only four of them adjusted for covariates. Failing to control for confounding factors can result in an over- or under-estimation of the true relationship between HF and cancer risk. Our sensitivity analysis reinforced this assertion, revealing that utilizing solely adjusted estimates led to a reduction in the hazard and a decrease in the strength of association for cancer incidence in HF. In fact, for haematological malignancies, the sensitivity analysis utilizing only adjusted estimates yielded non-significant trends. Therefore, the causal inference of this association remains debatable as there may be other correlated variables that may account for the observed link between HF and haematological or lung cancer.

Besides these gross methodological differences among the included studies, there were differences in the characteristics of the sample population as well. The countries and races in which the included studies were conducted differed, which may have important implications for the generalizability and interpretation of our results. The incidence, prevalence, and distribution of both HF and cancer may vary widely across different geographic regions and ethnic groups.^30,31^ For instance, some populations may have a higher incidence of specific types of cancer or HF, which may impact the overall association between HF and cancer risk. Moreover, differences in lifestyle factors, environmental exposures, and healthcare access across different countries and races may also contribute to disparities in the observed cancer incidence.^2,3,30^ Furthermore, there were notable differences in the gender and age distribution of the study populations. In particular, the studies by Selvaraj *et al*.^23^ that included only men and Leedy *et al*.^27^ that included only post-menopausal women may have important implications for the generalizability of our results.

Our subgroup analyses by study design revealed a slightly lower incidence of cancer among HF patients in prospective studies compared with retrospective studies. However, this difference was not statistically significant. This finding suggests that the lower risk observed in prospective studies may be due to inherent differences in the two study designs and methodology rather than actual differences in cancer incidence. Follow-up durations are another important factor to consider when assessing the risk of chronic conditions such as cancer. Shorter follow-up durations may not capture the full range of cancer incidence in patients with HF, while longer follow-up durations may increase the probability of detecting cancer incidence in both HF and control groups, leading to potential biases. However, our subgroup analyses did not reveal any significant differences between studies with a follow-up duration of at least 5 years and those with <5 years of follow-up. Nonetheless, longer follow-up durations may provide a more accurate representation of the true incidence of cancer in both the HF and control groups.

The results of our meta-regression analysis uncovered that diabetes mellitus might function as a potential positive effect modifier in the incidence of cancer amongst HF patients. However, the precise relationship between diabetes mellitus and cancer remains poorly understood, despite previous large-scale meta-analyses approximating a 25–40% increased risk of mortality from any form of cancer in those with diabetes mellitus.^32^ This association might be attributable to insulin secretion defects in diabetes mellitus that are linked to chronic inflammation, which is typified by high levels of oxidative stress, reactive oxygen species, and the activation of pro-inflammatory pathways.^33–35^ Furthermore, there is a complex inter-relationship between diabetes mellitus and HF, as individuals with diabetes mellitus are at an elevated risk of developing HF. This could be attributed to shared risk factors such as obesity, hypertension, and a sedentary lifestyle, as well as specific pathways that are altered in both conditions, including inflammation, oxidative stress, and endothelial dysfunction.^33,35^ Chronic inflammation has indeed been identified as a catalyst for tumour growth, enhanced metastasis, augmented angiogenesis, and reduced functionality of natural killer cells and macrophages.^8,29,36–38^

It is important to draw attention to the fact that none of the included studies took the severity of HF into account. Severe HF has been linked to biological changes such as chronic inflammation, immune system dysfunction, and oxidative stress, which increase cancer risk.^8,9,29,39^ Studies including patients with severe HF may observe a higher incidence of cancer in this group. Conversely, the severity of HF may also reduce cancer screening rates, resulting in an under-estimation of cancer incidence in the HF group. Family history of cancer is another critical factor that affects this association, but it was not accounted for in most studies. The study by Selvaraj *et al*.^23^ was the only exception, which adjusted hazards for a family history of cancer. Individuals with a family history of cancer may have shared genetic or environmental factors that increase cancer risk. Moreover, these individuals are also more likely to get screened for cancer leading to earlier detection rates.

In conclusion, our meta-analysis provides important insights into the relationship between HF and the incidence of cancer and several cancer subtypes, including breast cancer, lung cancer, haematological cancer, and colorectal cancer, adding to the growing body of evidence that suggests a potential link between these conditions. Our meta-regression analysis identified diabetes mellitus as a potential positive effect modifier, which highlights the need to consider comorbidities when assessing cancer risk in HF patients. The intriguing concept of HF acting as a trigger for cancer warrants further exploration. The results of this meta-analysis have important clinical implications, highlighting the need for increased cancer screening and prevention measures in HF patients. Current cardio-oncology guidelines by the European Scientific Society and American Heart Association include recommendations on pharmacokinetic considerations in the cardiovascular treatment regimens in cancer patients and cancer treatment regimens in cardiovascular patients.^40,41^ These guidelines also highlight the significance of interdisciplinary communication and expertise in cardiology, oncology, and haematology.^40^ Future research should focus on elucidating the underlying mechanisms linking HF and cancer, identifying effective screening and prevention strategies, and developing tailored approaches to the management of this vulnerable patient population.

### Strength and limitations

The key strength of this meta-analysis lies in the comprehensive evaluation of the overall incidence of cancer and the coverage of wide arrays of types of cancer classified by organ type or systems involved. Nonetheless, the study is limited by its reliance on observational studies, which may introduce confounding biases. While high heterogeneity was noted in several outcomes, the direction of effect sizes was largely consistent, although their magnitude varied across studies. In addition, although the pooled analysis suggested that there was a significant association between HF and cancer, the effect size could not be precisely quantified, perhaps due to variations in baseline patient characteristics and sample sizes.

Of note, there appeared to be a lack of association between HF and the incidence of prostate cancer. Further interpretation of current data revealed that only four studies were included in the analysis and that the rate of prostate cancer screening also differed across studies. For example, the study by Selvaraj *et al*.^23^ had a relatively low prostate-specific antigen screening rate among men during the follow-up period. Therefore, further research with a larger sample size and standardized screening procedures for prostate cancer would be necessary to confirm or refute the observed lack of correlation between HF and prostate cancer.

Furthermore, it was unclear whether the association of HF and the incidence of various types of cancer differs by the subtypes of HF, namely HFrEF and HFpEF, due to the lack of available data. It is worth noting that distinguishing between these subtypes could be of utmost importance, considering that HFpEF is frequently linked with multiple comorbidities, such as hypertension, chronic kidney disease, chronic obstructive pulmonary disease, and diabetes, each of which independently increases the likelihood of developing cancer.^8^ This factor can act as a confounding variable, affecting the results. The study by Leedy *et al*.^27^ was the only one to conduct a stratified analysis based on left ventricular ejection fraction, and their findings indicated that HFpEF is more closely associated with overall cancer incidence, as well as the occurrence of lung and colorectal cancers, compared with HFrEF. Therefore, future investigations should strive to gather more data on this topic and conduct more robust analyses to examine the relationship between HF and the incidence of various cancer types across the two subtypes.

Additionally, the use of imaging with radiation exposure, such as coronary computed tomography (CT), coronary angiography, and nuclear cardiac imaging, may be a potential risk factor for cancer development in HF patients. Conversely, imaging or therapy with radiation exposure, such as positron emission tomography, CT, and radiotherapy, may be necessary for oncological patients. The impact of these variables on HF patients, however, has yet to be explored. Therefore, further randomized controlled trials with large sample sizes and well-designed methodologies are required to elucidate these associations fully.

## Conclusions

Patients with HF had a higher incidence of cancer and several cancer subtypes, including breast, colorectal, lung, and haematological malignancies. Intensive screening for cancer in these populations may be warranted. Further studies should investigate the risk factors of cancer development in these patients, especially the use of imaging with radiation exposure in the specialized population.

## Lead author biography


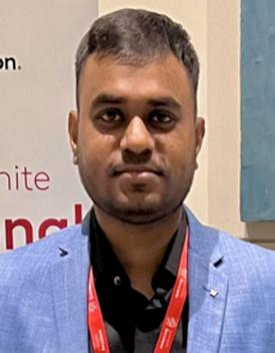
Vikash Jaiswal is a Doctor and outcome-based Cardiovascular researcher at Larkin Community Hospital, South Miami, FL, USA. Alongside, he runs his own JCCR cardiology research group, which has published quality papers in reputable journals. He is interested in exploring the field of Cardio-Oncology, preventive cardiology, and racial disparities among cardiovascular outcomes. He won the Prestigious Paul Dudley White International Scholar Award in 2021 and 2022 from American Heart Association for best Cardiovascular Research from the Philippines and Indian regions. His father and elder brother Dr Akash Jaiswal keep motivating him to perform his best towards humanity and in the field of medicine.

## Supplementary Material

oead073_Supplementary_Data

## Data Availability

The data underlying this article are available in the article and its online Supplementary material.
