# Supplementary material for: Association between heart failure and the incidence of cancer: a systematic review and meta-analysis
Source: Eur Heart J Open. 2023 Aug 3;3(5):oead073. doi: 10.1093/ehjopen/oead073 (PMC10561890; doi:10.1093/ehjopen/oead073)
Supplement: oead073_Supplementary_Data [file oead073_supplementary_data.docx]

**Supplementary materials**

**Supplementary Table 1:** Search strategy among different databases

**Supplementary Table 2:** Newcastle-Ottawa scale for quality assessment and bias assessment of observational studies.

**Supplementary Figure 1** PRISMA flow diagram

**Supplementary Figure 2** Leave-one-out analysis for incidence of cancer

**Supplementary Figure 3** Sensitivity analysis using adjusted estimates only for incidence of cancer.

**Supplementary Figure 4** Sensitivity analysis using adjusted estimates only for incidence of colorectal cancer.

**Supplementary Figure 5** Leave-one-out analysis for colorectal cancer

**Supplementary Figure 6** Leave-one-out analysis for breast cancer

**Supplementary Figure 7** Sensitivity analysis using adjusted estimates only for incidence of breast cancer.

**Supplementary Figure 8** Sensitivity analysis using adjusted estimates only for prostate cancer.

**Supplementary Figure 9** Sensitivity analysis using adjusted estimates only for lung cancer.

**Supplementary Figure 10** Leave-one-out analysis for lung cancer

**Supplementary Figure 11** Sensitivity analysis using adjusted estimates only for hematological cancer.

**Supplementary Figure 12** Leave-one-out analysis for hematological cancer

**Supplementary Figure 13** Subgroup analyses on the incidence of cancer based on study design.

**Supplementary Figure 14** Subgroup analyses on the incidence of cancer based on follow-up period.

**Supplementary Figure 15** Subgroup analyses on the incidence of cancer based on sample size.

**Supplementary Figure 16** Meta-regression of hypertension as a potential effect modifier on incidence of cancer

**Supplementary Figure 17** Meta-regression of diabetes mellitus as a potential effect modifier on incidence of cancer

**Supplementary Figure 18** Funnel plot of incidence of overall cancer

**Supplementary Figure 19** Funnel plot of breast cancer

**Supplementary Figure 20** Funnel plot of lung cancer

**Supplementary Figure 21** Funnel plot of hematological cancer**Supplementary Table 1:** Search strategy among different databases

**Supplementary Table 2:** Newcastle-Ottawa scale for quality assessment and bias assessment of observational studies.

**Supplementary Figure 1** PRISMA flow diagram

**Supplementary Table 1:** Search strategy among different databases

| **Electronic database** | **Search strategy** |
| --- | --- |
| PubMed | ((((((((Heart failure [MeSH Terms]) OR (Heart failure)) OR (cardiac dysfunction[Other Term])) OR (Myocardial Ischemia[Other Term])) AND (Cancer[MeSH Terms])) OR (Cancer[Other Term])) OR (Malignancy[Other Term])) OR (Neoplasm[MeSH Terms])) OR (Neoplasm[Other Term]) |
| Embase | “Heart Failure” AND “Cancer” AND “Neoplasm” AND “Malignancy” |
| Scopus | “Heart Failure” AND “Cancer” AND “Neoplasm” AND “Malignancy” |

**Supplementary Table 2**. Newcastle-Ottawa scale for quality assessment and bias assessment of observational studies.

| **Study** | **Selection** | | | | **Comparability** | **Outcome** | | | **Total*** |
| --- | --- | --- | --- | --- | --- | --- | --- | --- | --- |
|  | **Representatives of exposed group** | **Selection of non-exposed cohort** | **Ascertainment of exposure** | **Outcome of interest** |  | **Outcome assessment** | **Adequacy of follow up duration** | **Adequacy of follow up of cohort** |  |
| [Kwak et al](https://pubmed.ncbi.nlm.nih.gov/32863081/), 2020 | 1 | 1 | 1 | 1 | 2 | 1 | 1 | 1 | 9 |
| [Hasin et al](https://pubmed.ncbi.nlm.nih.gov/23810869/), 2013 | 1 | 1 | 1 | 1 | 1 | 1 | 1 | 1 | 8 |
| [Banke et al](https://pubmed.ncbi.nlm.nih.gov/26751260/), 2016 | 1 | 1 | 1 | 1 | 2 | 1 | 1 | 1 | 9 |
| [Hasin et al](https://www.ncbi.nlm.nih.gov/pmc/articles/PMC4947209/), 2017 | 1 | 1 | 1 | 1 | 1 | 1 | 1 | 1 | 8 |
| [Selvaraj et al](https://pubmed.ncbi.nlm.nih.gov/29622155/), 2018 | 1 | 1 | 1 | 1 | 2 | 1 | 1 | 1 | 9 |
| [Schwartz et al](https://pubmed.ncbi.nlm.nih.gov/32446924/), 2020 | 1 | 1 | 1 | 1 | 2 | 1 | 1 | 1 | 9 |
| [Roderburg et al](https://www.ncbi.nlm.nih.gov/pmc/articles/PMC8497216/), 2021 | 1 | 1 | 1 | 1 | 0 | 1 | 1 | 1 | 7 |
| [D.J. Leedy et al](https://onlinelibrary.wiley.com/doi/abs/10.1002/ejhf.2207), 2021 | 1 | 1 | 1 | 1 | 1 | 1 | 1 | 0 | 7 |
| [Bertero et al](https://www.sciencedirect.com/science/article/pii/S2666087321003859?via%3Dihub), 2022 | 1 | 1 | 1 | 1 | 2 | 1 | 1 | 1 | 9 |

  *Score >6 was considered as an adequate quality study.


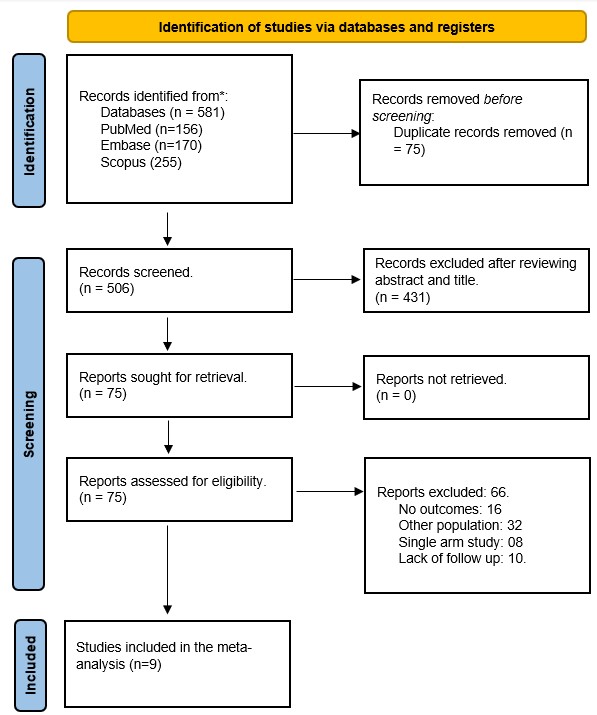


**Supplementary Figure 1** PRISMA flow diagram


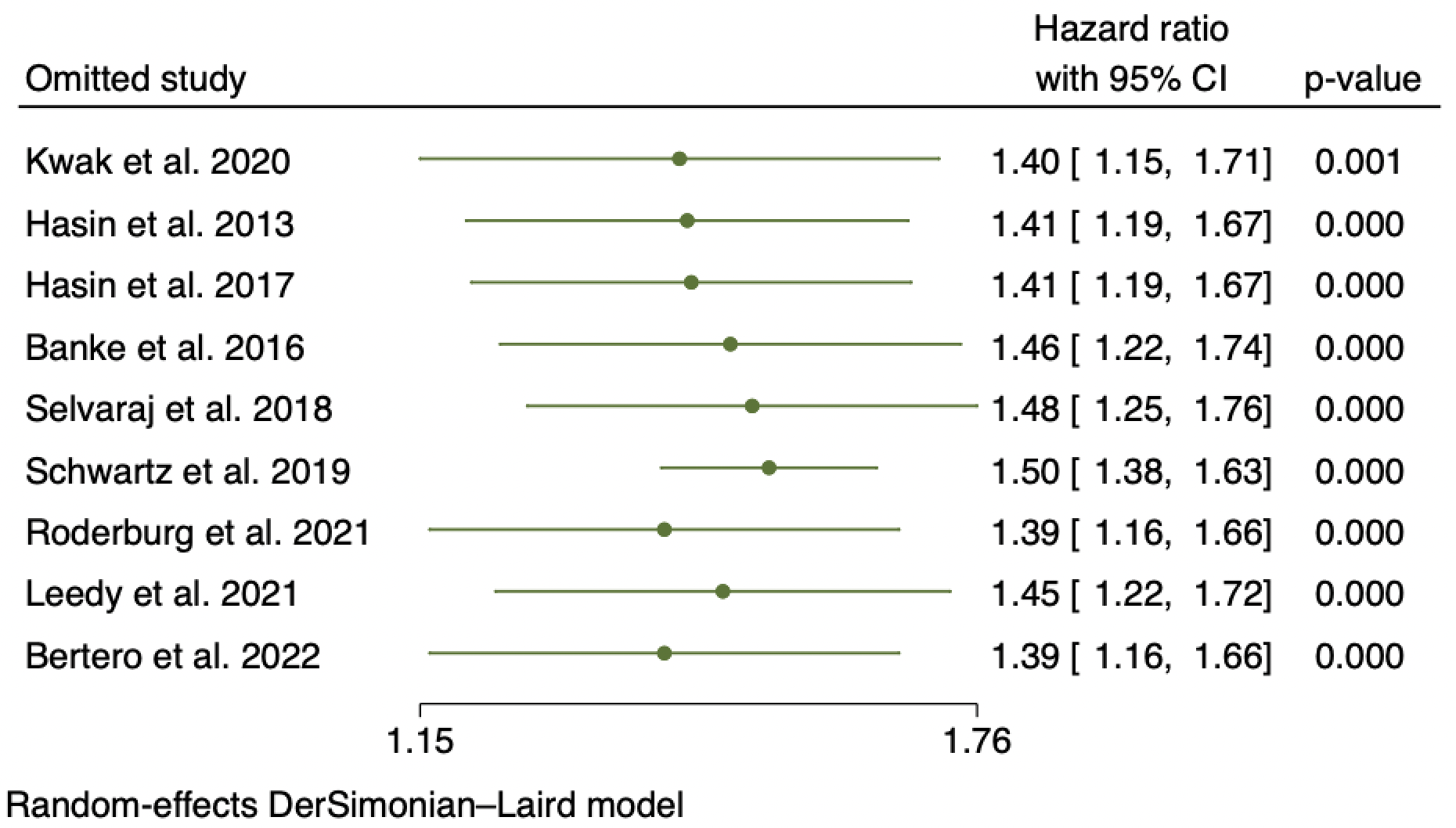


**Supplementary Figure 2** Leave-one-out analysis for incidence of cancer


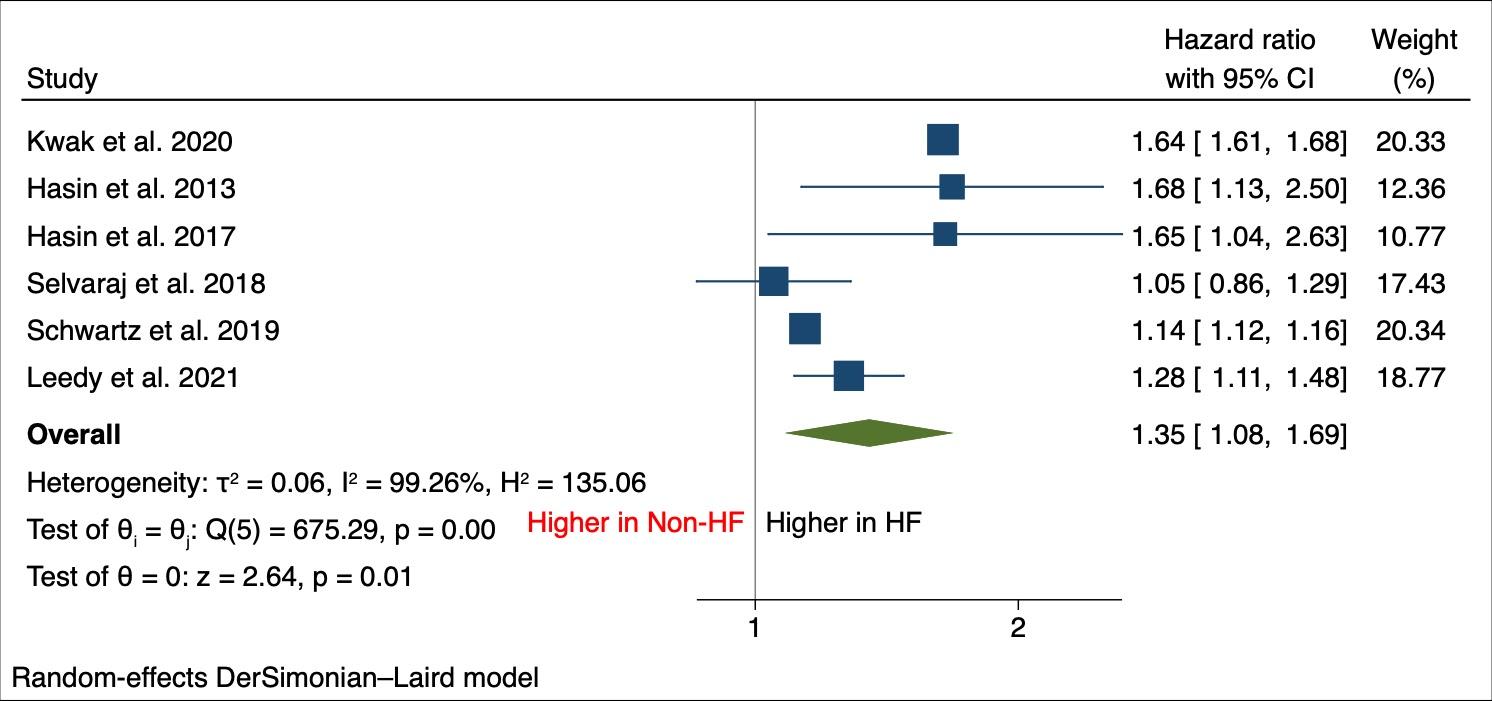


**Supplementary Figure 3** Sensitivity analysis using adjusted estimates only for incidence of cancer.


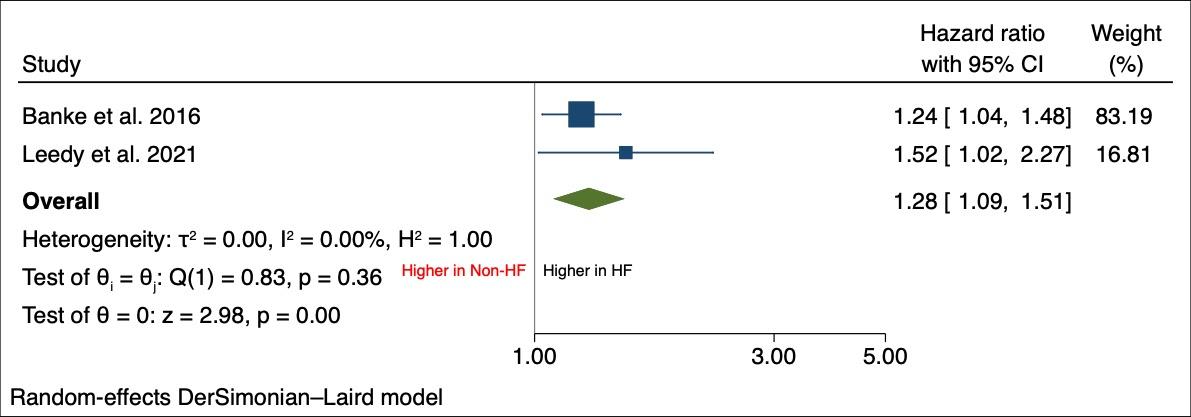


**Supplementary Figure 4** Sensitivity analysis using adjusted estimates only for incidence of colorectal cancer.


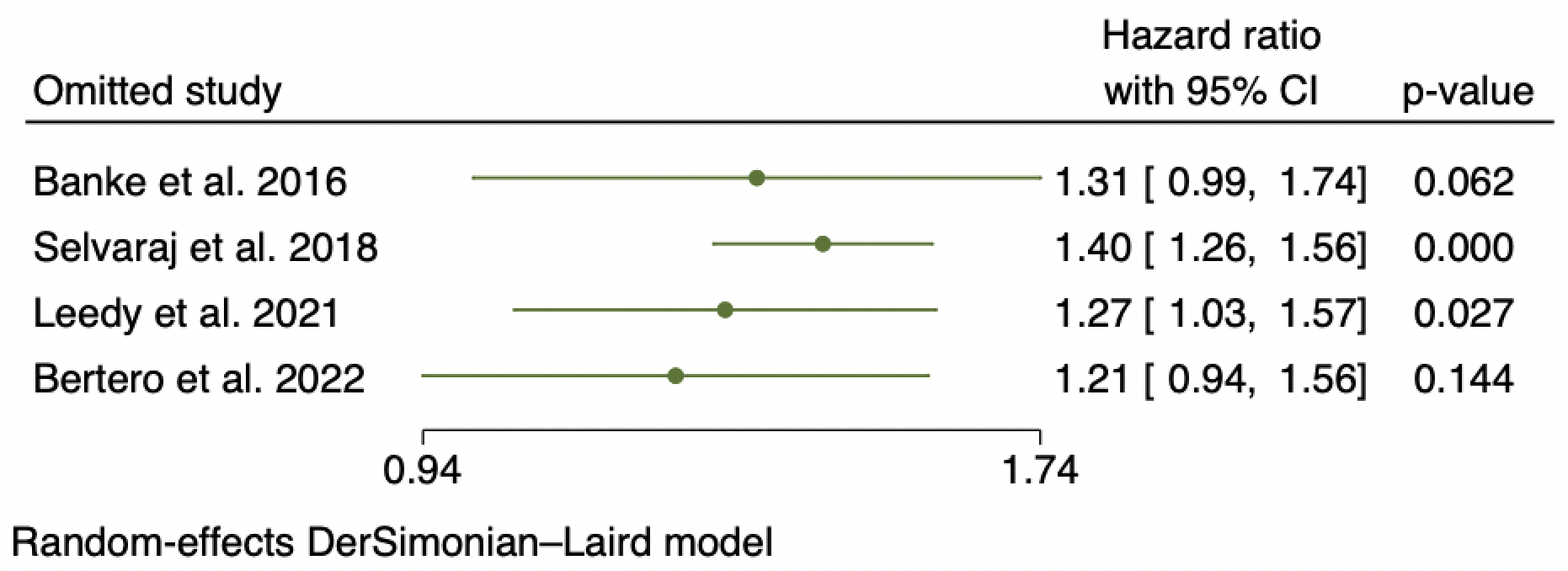


**Supplementary Figure 5** Leave-one-out analysis for colorectal cancer


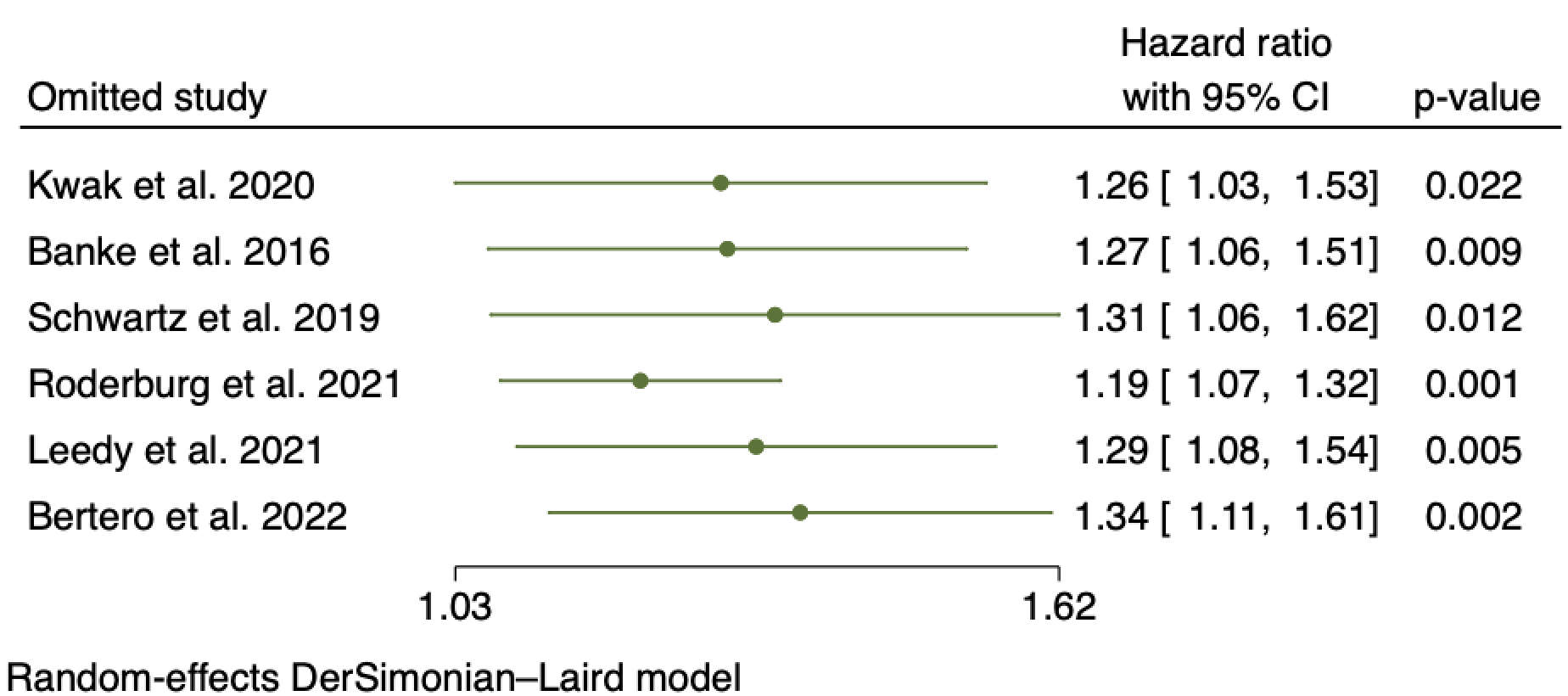


**Supplementary Figure 6** Leave-one-out analysis for breast cancer

**
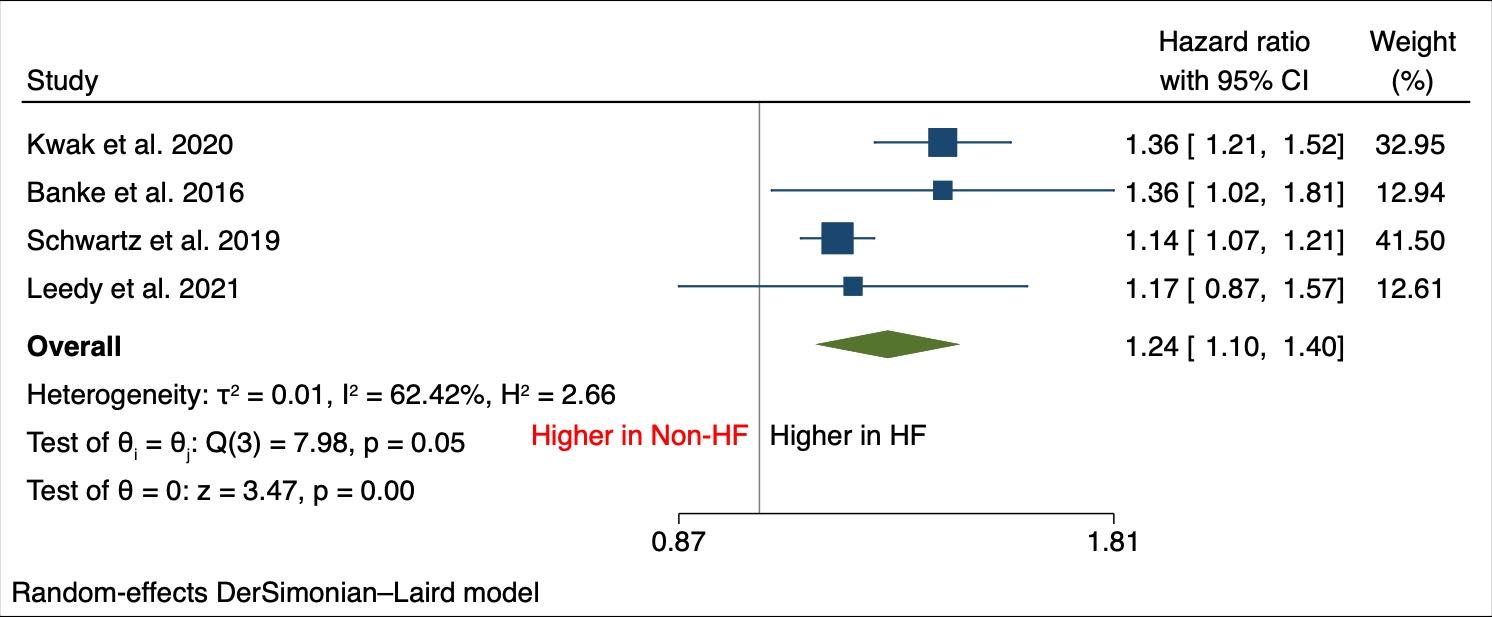
**

**Supplementary Figure 7** Sensitivity analysis using adjusted estimates only for incidence of breast cancer.


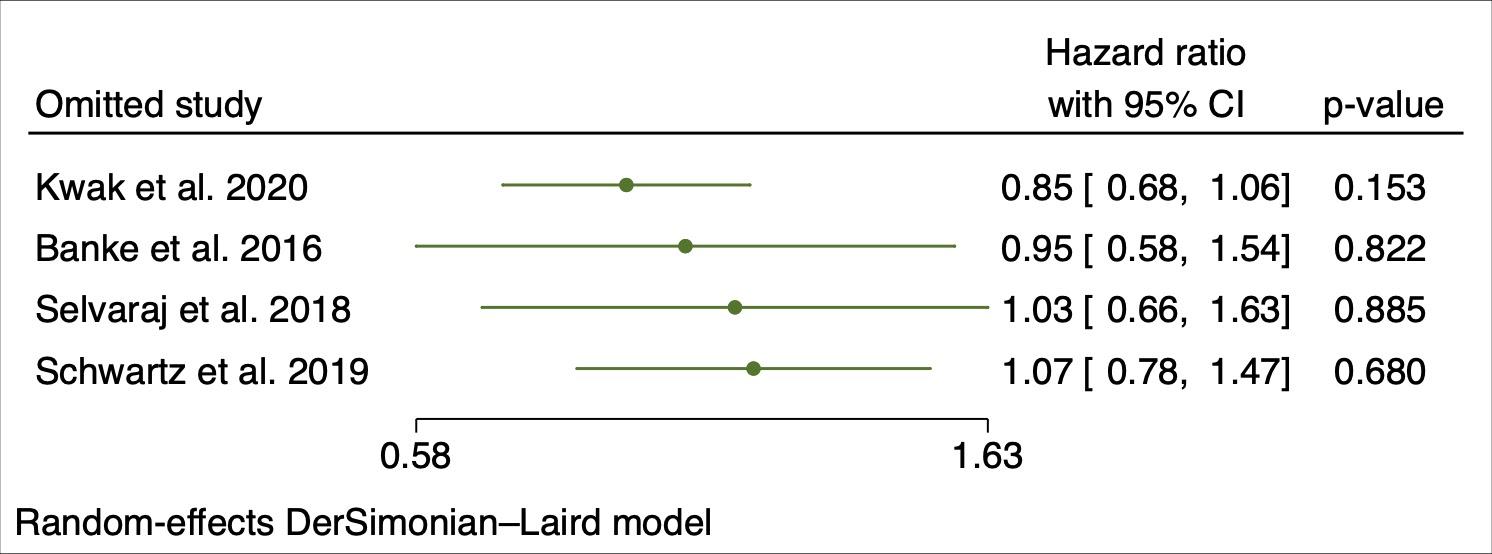


**Supplementary Figure 8** Leave-one-out analysis for prostate cancer

**
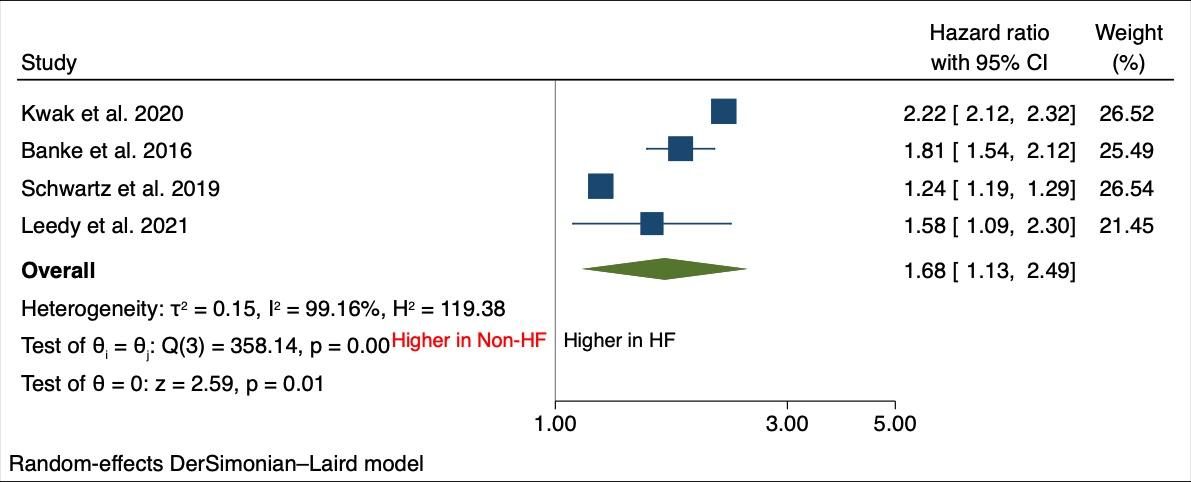
**

**Supplementary Figure 9** Sensitivity analysis using adjusted estimates only for lung cancer.


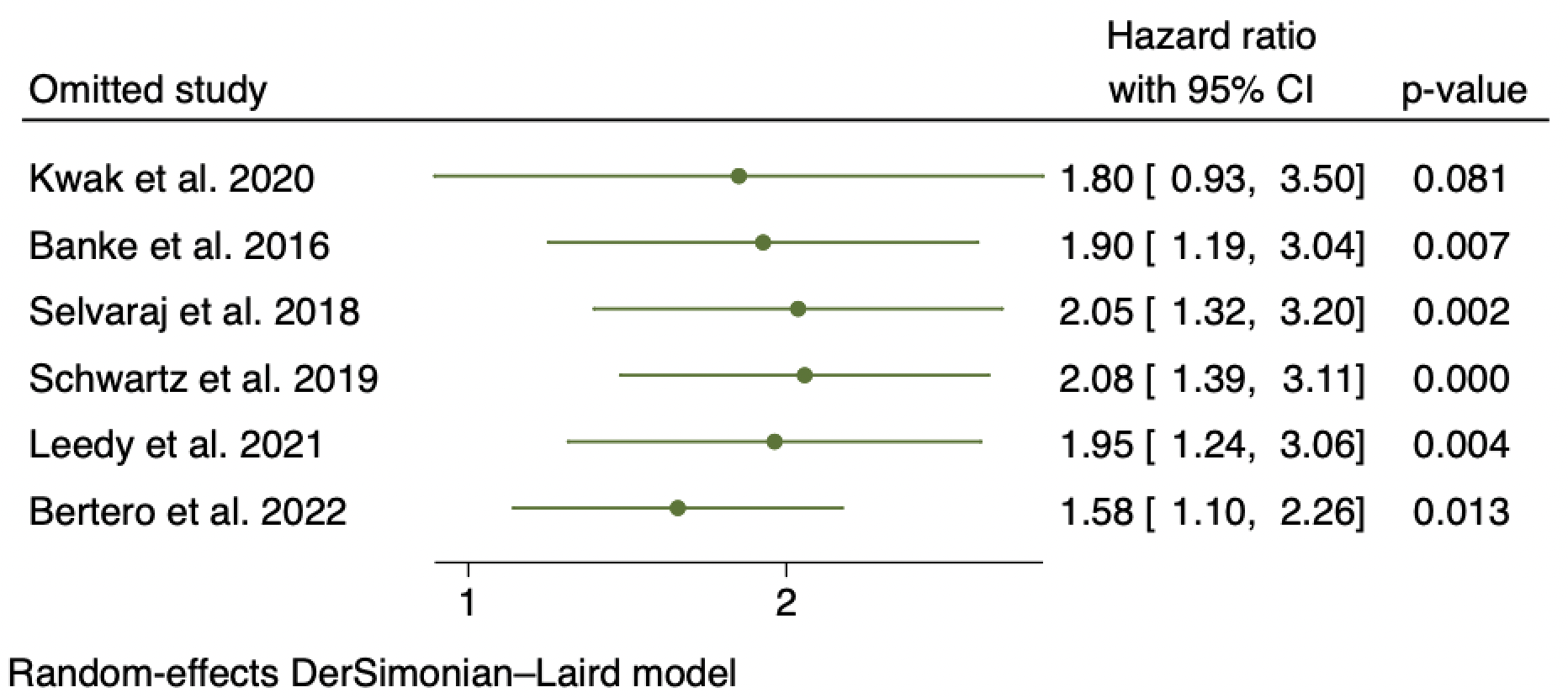


**Supplementary Figure 10** Leave-one-out analysis for lung cancer.


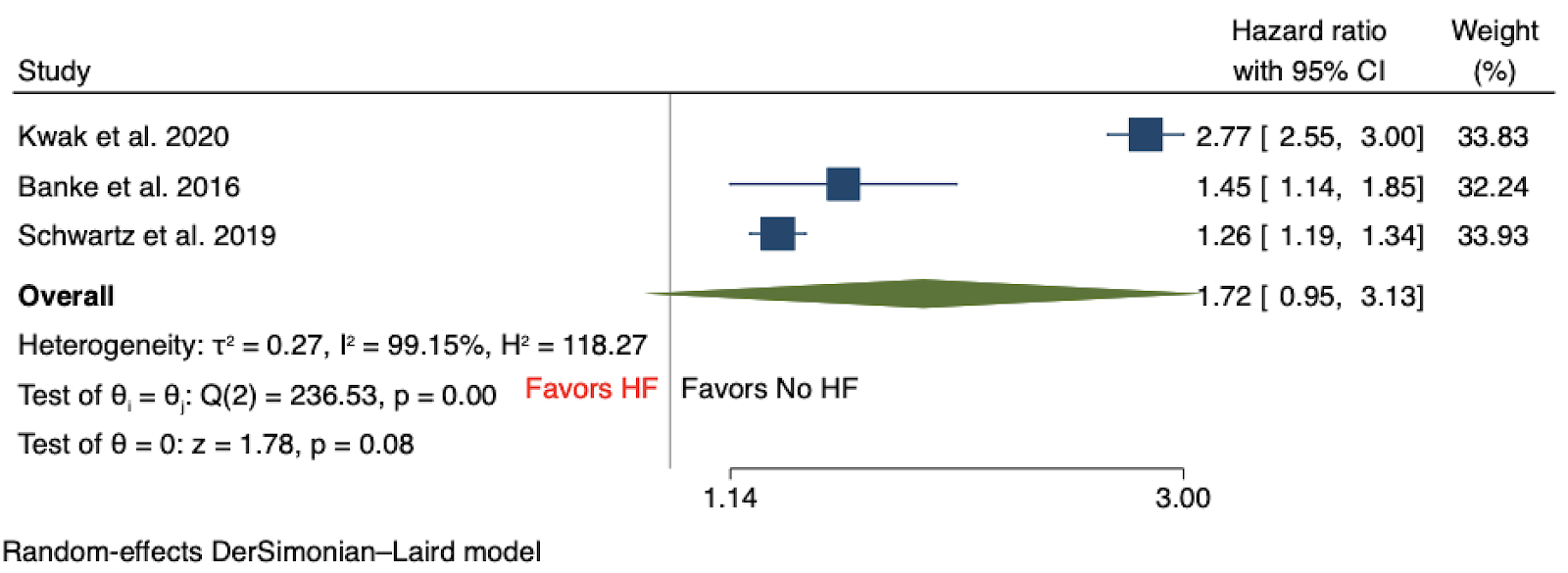


**Supplementary Figure 11** Sensitivity analysis using adjusted estimates only for hematological cancer.

**
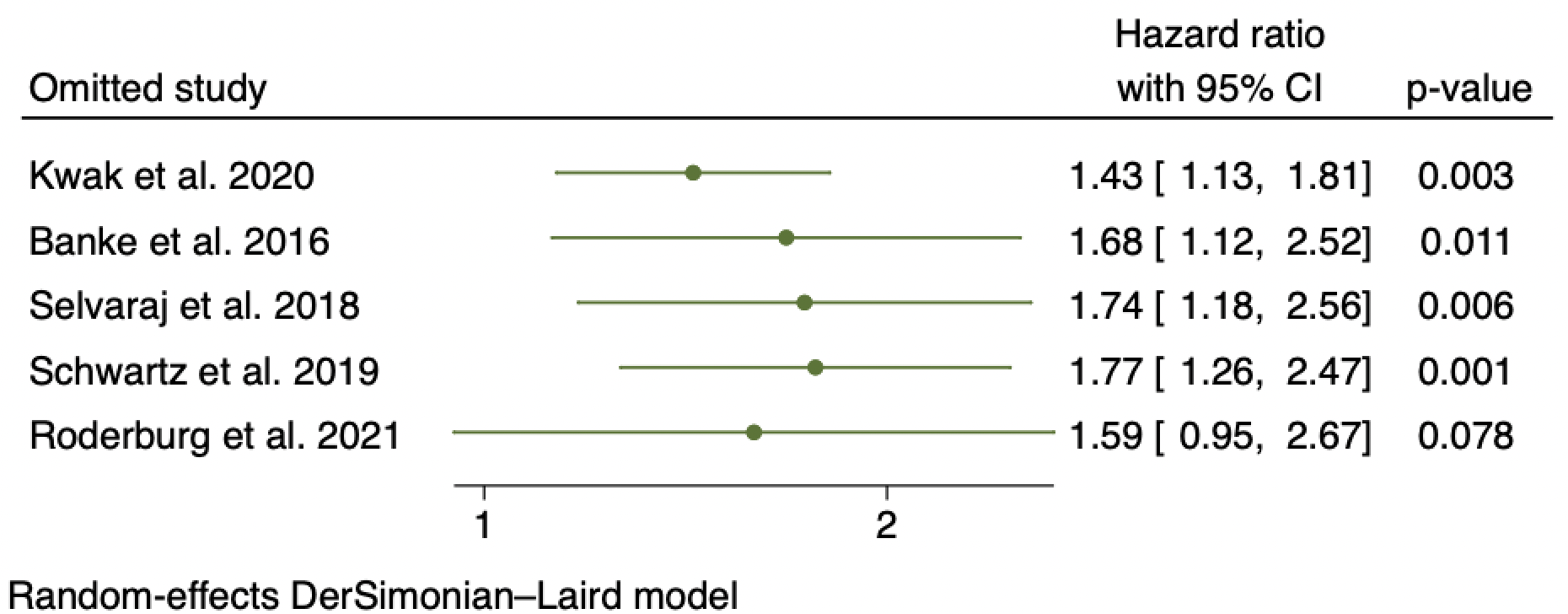
**

**Supplementary Figure 12** Leave-one-out analysis for hematological cancer


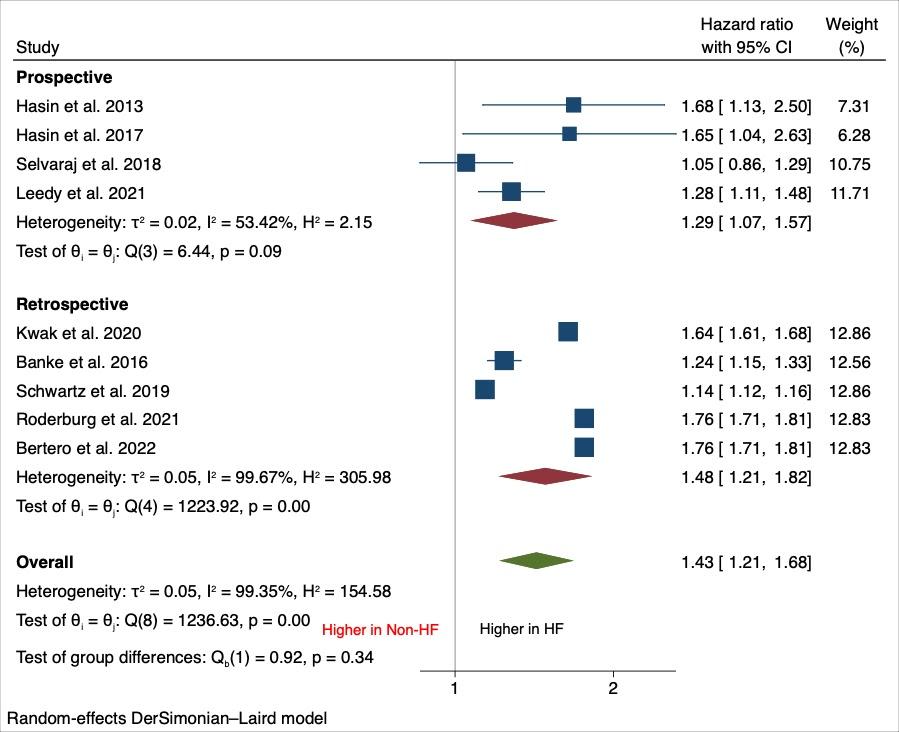


**Supplementary Figure 13** Subgroup analyses on incidence of cancer based on study design


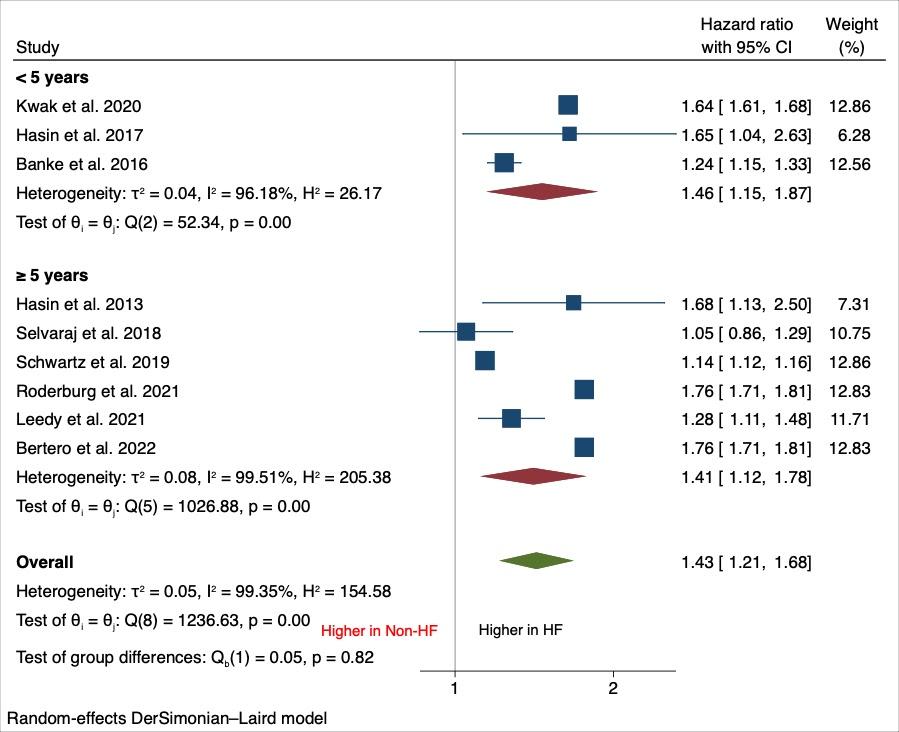


**Supplementary Figure 14** Subgroup analyses on incidence of cancer based on follow-up period

**
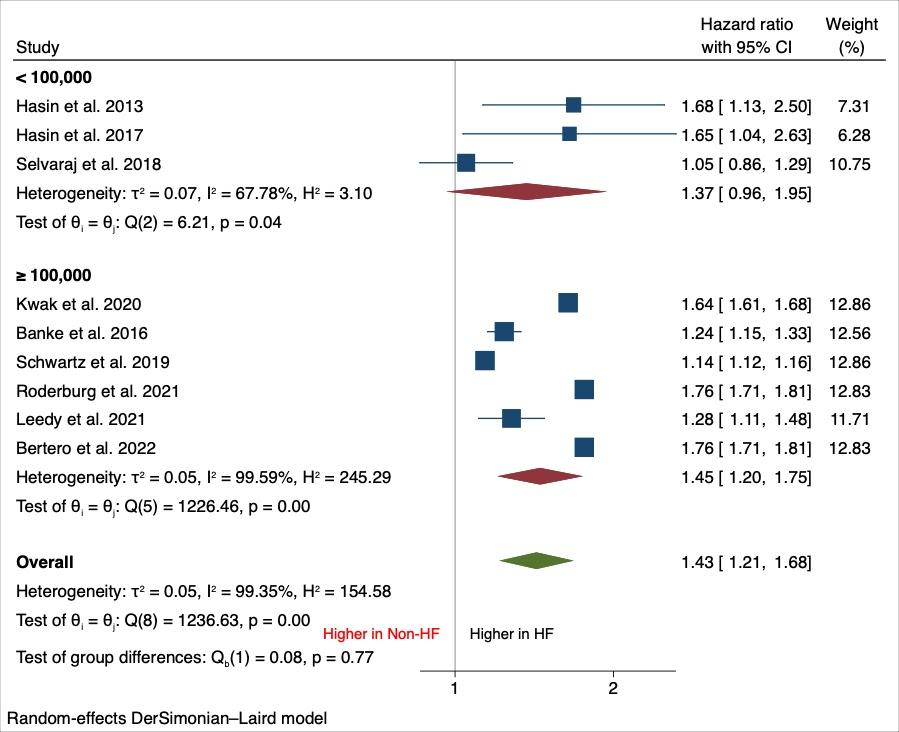
**

**Supplementary Figure 15** Subgroup analyses on incidence of cancer based on sample size

**
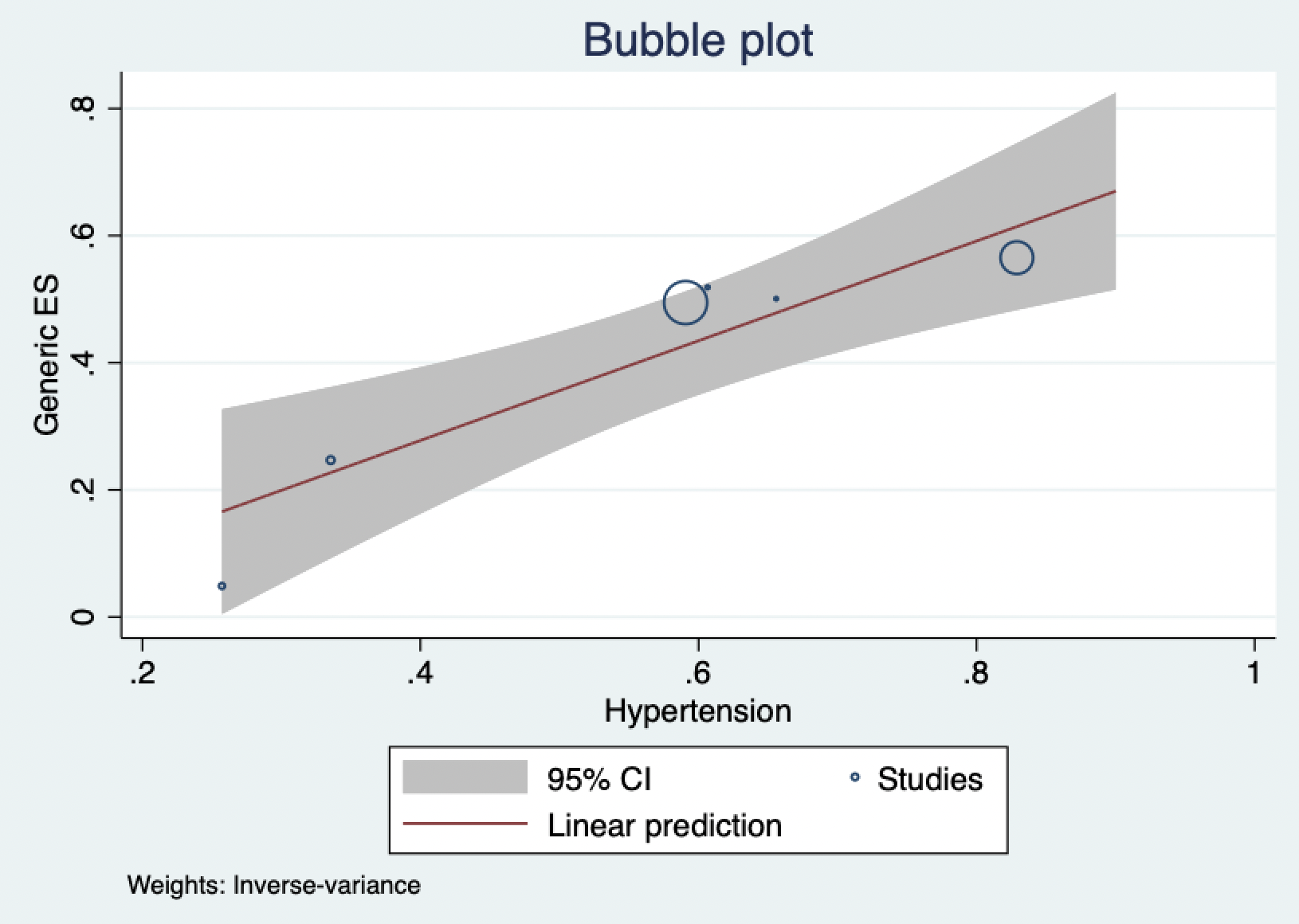
**

**Supplementary Figure 16** Meta-regression of hypertension as a potential effect modifier on incidence of cancer

**
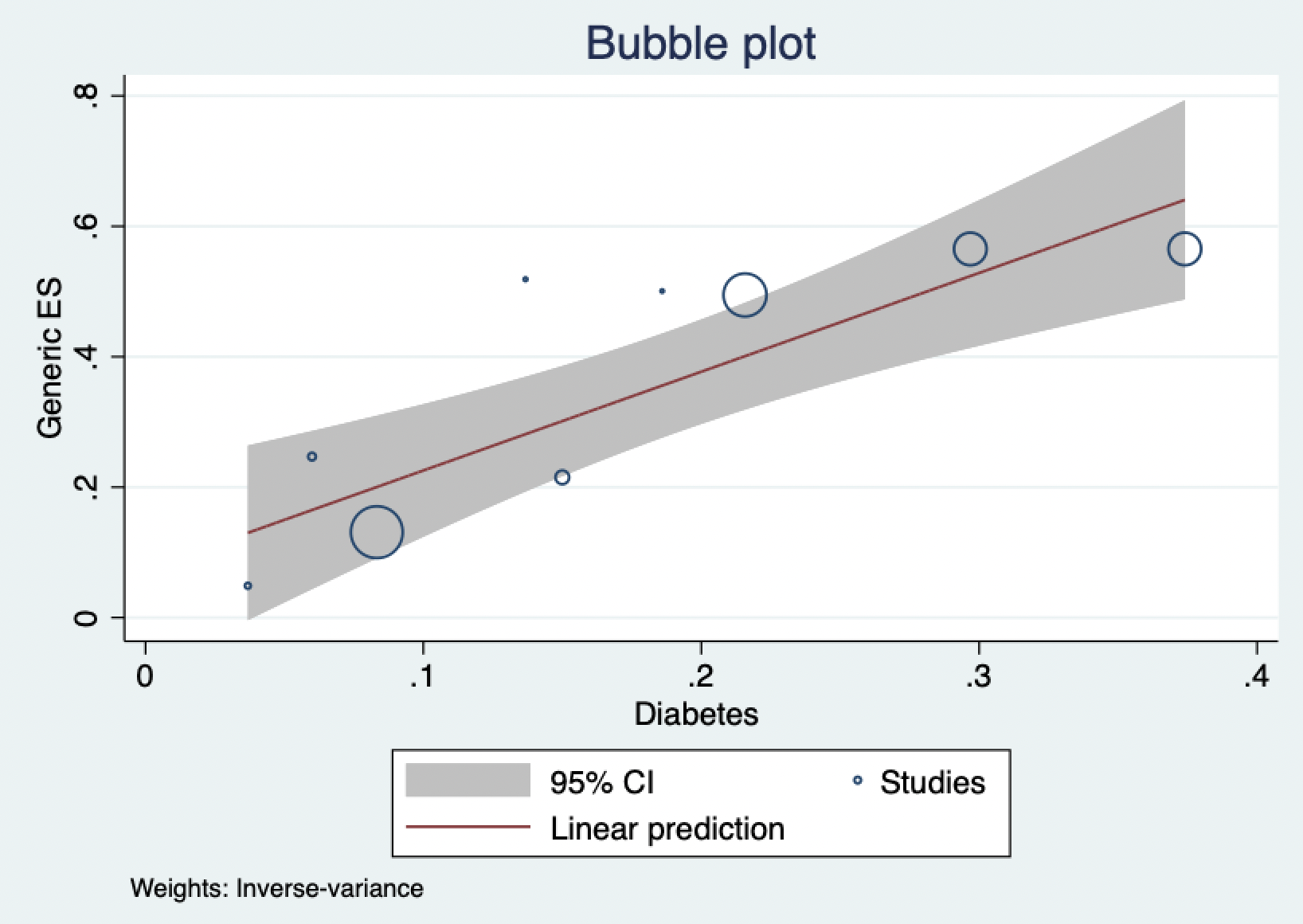
**

**Supplementary Figure 17** Meta-regression of diabetes mellitus as a potential effect modifier on incidence of cancer

**
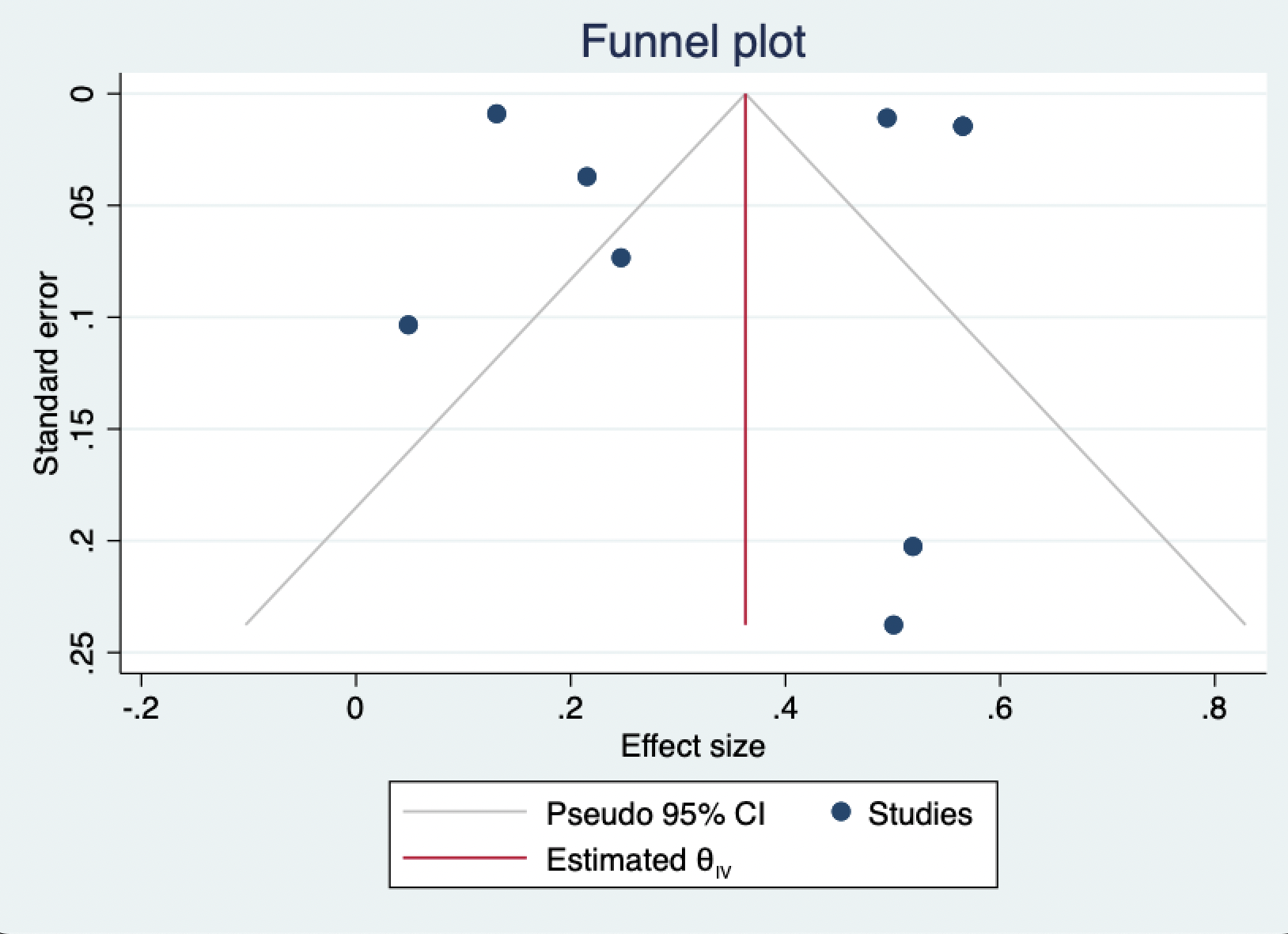
**

**Supplementary Figure 18** Funnel plot of incidence of overall cancer


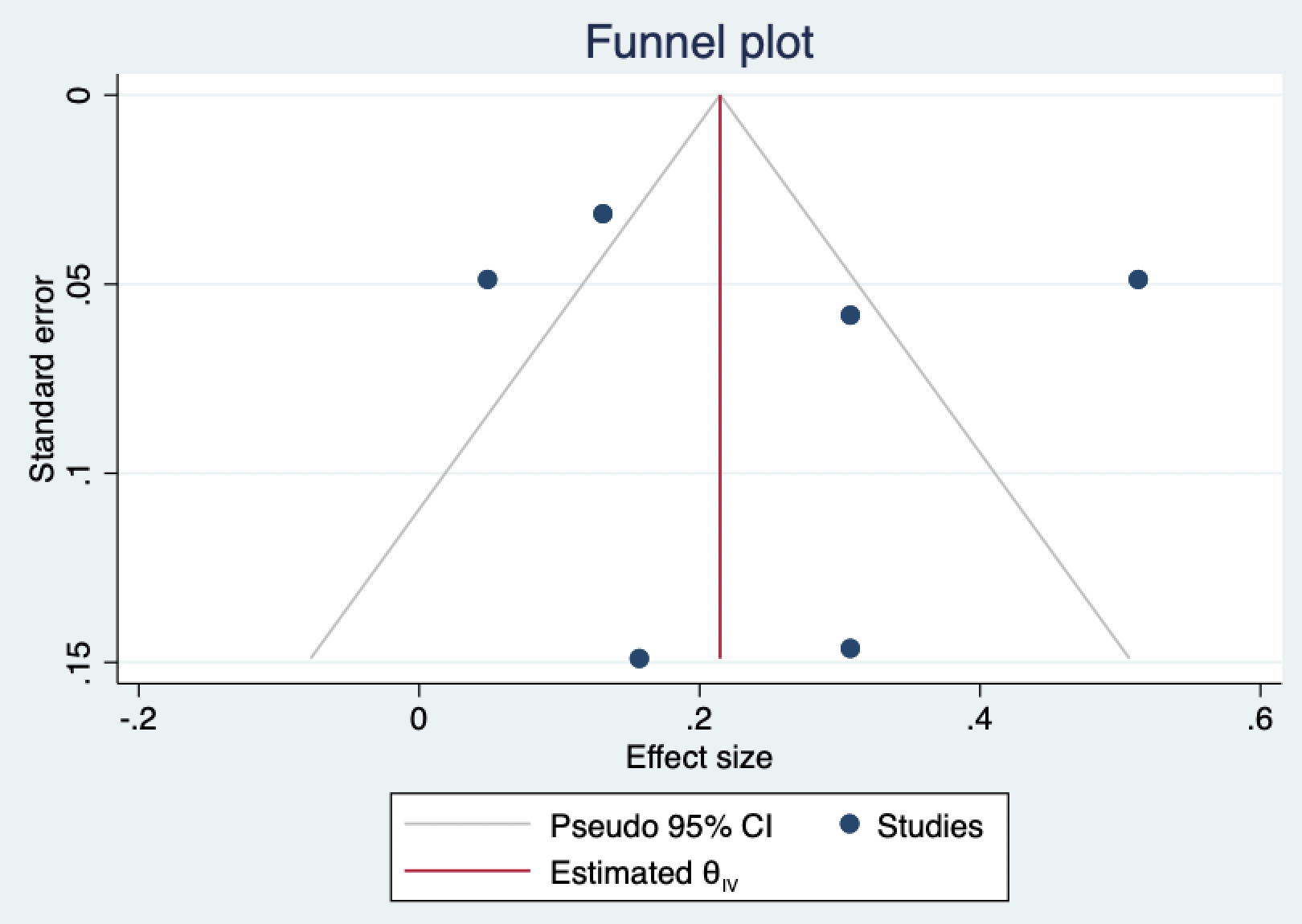


**Supplementary Figure 19** Funnel plot of breast cancer


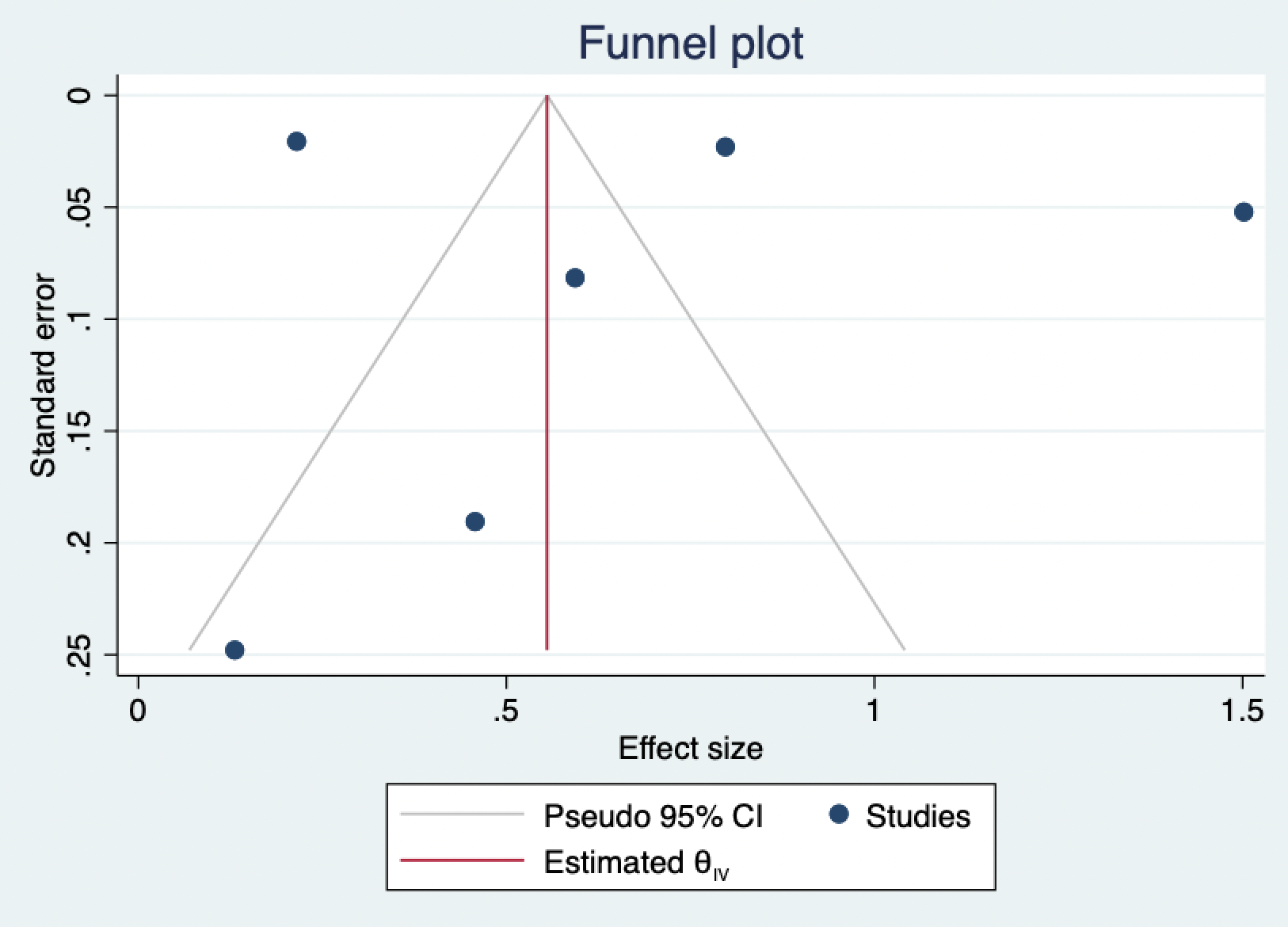


**Supplementary Figure 18** Funnel plot of lung cancer

**
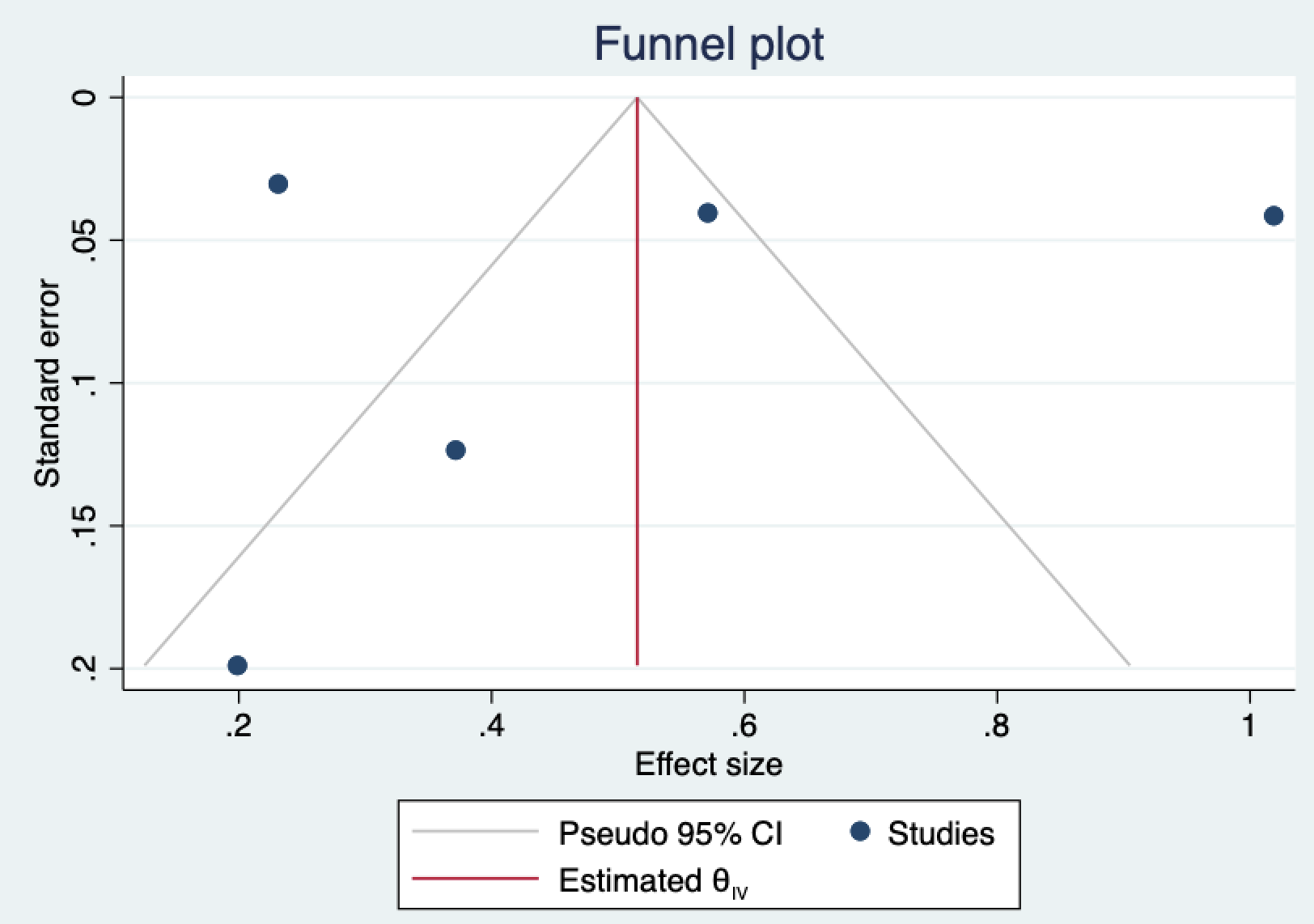
**

**Supplementary Figure 18** Funnel plot of hematological cancer
